# 17β-Estradiol promotes metastasis in triple-negative breast cancer through the Calpain/YAP/β-catenin signaling axis

**DOI:** 10.1371/journal.pone.0298184

**Published:** 2024-03-28

**Authors:** Xuemei Niu, Jianan Wang, Jinguang Liu, Qinglong Yu, Mingwei Ci

**Affiliations:** 1 Department of oncology, Weihai Central Hospital, Weihai, China; 2 Yantai Key Laboratory of Nanomedicine & Advanced Preparations, Yantai Institute of Materia Medica, Yantai, Shandong, China; Universita degli Studi della Campania Luigi Vanvitelli, ITALY

## Abstract

β-catenin is an important regulator of malignant progression. 17β-Estradiol (E2), an important sex hormone in women, promotes the growth and metastasis of triple-negative breast cancer (TNBC). However, whether β-catenin is involved in E2-induced metastasis of TNBC remains unknown. In this study, we show that E2 induces the proliferation, migration, invasion, and metastasis of TNBC cells. E2 induces β-catenin protein expression and nuclear translocation, thereby regulating the expression of target genes such as Cyclin D1 and MMP-9. The inhibition of β-catenin reversed the E2-induced cell malignant behaviors. Additionally, E2 activated Calpain by increasing intracellular Ca^2+^ levels and reducing calpastatin levels. When Calpain was inhibited, E2 did not induce the proliferation, migration, invasion, or metastasis of TNBC cells. In addition, E2 promoted translocation of YAP into the nucleus by inhibiting its phosphorylation. Calpain inhibition reversed the E2-induced YAP dephosphorylation. Inhibition of YAP transcriptional activity reversed the effects of E2 on the proliferation, migration, invasion, and β-catenin of TNBC cells. In conclusion, we demonstrated that E2 induced metastasis-related behaviors in TNBC cells and this effect was mediated through the Calpain/YAP/β-catenin signaling pathway.

## Introduction

Breast cancer is a heterogeneous disease with multiple subtypes and various clinical features. Triple-negative breast cancer (TNBC) is an aggressive subtype that accounts for 15%–20% of all breast cancers [1]. As TNBC cells lack estrogen receptor (ER), progesterone receptor, and human epidermal growth factor receptor-2, targeted therapy is not effective. Therefore, the main treatments for TNBC are surgery, chemotherapy, and radiotherapy [2, 3]. TNBC is prone to recurrence and metastasis, with low overall and disease-free survival rates [2, 3]. 17β-Estradiol (E2), an important sex hormone in women, is involved in growth and development and maintaining female secondary sex characteristics. E2 exerts its biological effects mainly through the ER [4, 5]. Under pathological conditions, E2 promotes the malignant progression of hormone-dependent tumors by enhancing ER-positive breast cancer cell proliferation [6, 7], invasion [6], and metastasis [8]. However, recent studies have shown that E2 also promotes the proliferation [9] and metastasis [10] of TNBC cells. Therefore, strategies aimed at inhibiting the cancer-promoting effects of E2 are clinically significant.

β-catenin, a key molecule in the classical Wnt signaling pathway, plays an important role in tumor proliferation and differentiation as well as stemness regulation [11, 12]. It interacts with the cytoskeleton to participate in extracellular signaling and can enter the nucleus to regulate target gene expression [11, 12]. β-catenin is expressed in TNBC cells and is involved in tumorigenesis and progression. In addition, β-catenin mediates TNBC cell metastasis induced by various signaling pathways, such as TUFT1/Rac1 [13], circPSMA1/miR-637/Akt1 [14], and ITGB1/FAK/Src/AKT [15]. Although E2 has been shown to regulate β-catenin expression and activation [16, 17], the role and potential mechanism of β-catenin in E2-induced TNBC cell metastasis remains unclear and require further investigation.

## Materials and methods

### Cell counting kit-8 assay

BT-549 or MDA-MB-231 cells were inoculated in 96-well plates at a density of 6.0 × 10^3^/well and incubated for 24 h. Subsequently, the cells were treated with 0, 1, 3, 10, 30, or 100 nM E2 (Sigma-Aldrich, St. Louis, MO, USA) for 48 h. To assess cell proliferation, Cell counting kit-8 (CCK-8) solution (10 μL) was added to each well and incubated for 4 h. The absorbance was then measured at 450 nm using a SpectraMax M2e microplate reader (Sartorius, Gottingen, Germany).

### Wound-healing experiment

Cells were seeded in 12-well plates at a density of 5 × 10^5^/well and cultured. When the cell density reached > 95% confluence, the cell monolayer was scratched using a pipette tip, and the plate was washed to remove non-adherent cells. The cells were treated with 30 nM E2 for 48 h after pretreatment with calpeptin (Calpain inhibitor), Super-TDU (YAP inhibitor), or ICG-001 (β-catenin inhibitor) (MedChem Express, Monmouth Junction, NJ, USA) for 4 h. The cell monolayer scratches were photographed at 0 and 48 h using a TS2 inverted microscope (Olympus, Tokyo, Japan).

### Invasion assay

An invasion chamber (8.0 μm; Corning, Corning, NY, USA) was prepared by adding 100 μL of 10-fold diluted Matrigel (Corning) to the upper chamber. Subsequently, the invasion chamber was incubated for 4 h. Cells pretreated with calpeptin, Super-TDU, or ICG-001 for 4 h were inoculated into the upper chamber. DMEM containing 30% fetal bovine serumwas added to the lower chamber. After treatment with 30 nM E2 for 48 h, the cells in the upper chamber were removed. The cells invading the lower chamber were fixed with 4% paraformaldehyde, stained with 0.1% crystal violet (Solarbio Life Sciences, Beijing, China), and then imaged using a TS2 inverted microscope.

### Western blotting

Total cellular and nuclear proteins were extracted using a protein extraction kit (Solarbio Life Sciences) and quantified using a protein quantification kit (Solarbio Life Sciences). The extracted total proteins were separated via sodium dodecyl sulfate–polyacrylamide gel electrophoresis and transferred to polyvinylidene fluoride membranes (PVDF; Thermo Fisher Scientific, Waltham, MA, USA). The membranes were sequentially incubated with blocking solution and primary and secondary antibodies, followed by luminescent signal detection using an enhanced chemiluminescence kit and imaging using an Amersham Imager 600 (GE, Chicago, IL, USA). The following primary antibodies were used: anti-β-catenin (1:1000), anti-Cyclin D1 (1:2000), anti-MMP-9 (1:1000), anti-calpastatin (1:1000), anti-p-YAP (1:1000), anti-YAP (1:1000), and anti-Lamin B1 (1:1000) (Cell Signaling Technology, Danvers, MA, USA); anti-GAPDH (1:3000), anti-p-LATS1 (1:1000), and anti-LATS1 (1:1000) (Cloud-Clone Corp., China). The secondary antibodies were goat anti-rabbit IgG-horseradish peroxidase (HRP) and goat anti-mouse IgG-HRP (Cell Signaling Technology).

### Immunofluorescence assays

Coverslips were placed in 12-well plates, inoculated with cells at a density of 1 × 10^5^/well, and incubated overnight. Subsequently, the cells were treated with E2 (30 nM) for 48 h, fixed with 4% paraformaldehyde, and permeabilized with a cell immunopenetrant (Solarbio Life Sciences). The cells were blocked with 3% goat serum and incubated overnight with primary antibody at 4°C. The following day, the cells were incubated with a fluorescent secondary antibody. Then, the cells were blocked with a fluorescence antifade solution containing DAPI. Imaging was performed using a Ti-U fluorescence inverted microscope (Nikon, Tokyo, Japan). The primary antibodies used were as follows: anti-β-catenin (1:200), anti-Cyclin D1 (1:500), anti-MMP-9 (1:100), and anti-YAP (1:100). The fluorescent secondary antibodies used were goat anti-rabbit IgG (H+L) (Alexa Fluor 488) (1:200) and goat anti-mouse IgG Fc (TRITC) (1:200).

### Calpain activity assay

Calpain activity was measured using a Calpain activity assay kit (Abcam, Cambridge, MA, USA) according to the manufacturer’s instructions. The absorbance at excitation/emission (Ex/Em) wavelengths of 400 nm/505 nm was measured using a microplate reader.

### Animal experiments

The animal experiments were approved by the Committee on Ethics of Weihai Central Hospital (T20230315) and performed in strict accordance with the Declaration of Helsinki. After 1 week of rearing, 5-week-old female NOD-SCID mice (GemPharmatech, Ltd., China) were divided into four groups: Control, E2, E2 + calpeptin, and E2 + ICG-001. The ovaries of the mice in each group were removed, and the animals were allowed to recover for one week. Subsequently, BT-549 cells (1.6 × 10^6^) were injected into the tail vein (as two separate doses). Extended-release estrogen tablets (0.36 mg) were implanted subcutaneously in all groups, except the Control group. The E2 + calpeptin group was administered calpeptin (2 mg/kg) twice weekly, and the E2 + ICG-001 group was administered ICG-001 (5 mg/kg) three times weekly. After 9 weeks, tumor-bearing mice were euthanized with deep anesthesia followed by cervical dislocation. To minimize discomfort, euthanasia was performed quickly. The lung tissues were dissected and fixed in 10% neutral formalin for analysis. All handling of mice was carried out in a gentle to minimize animal suffering and distress.

### Histological analysis

Paraffin-embedded lung tissues were sliced into 5-μm thick sections and stained using a hematoxylin and eosin (H&E) staining kit (Solarbio Life Science) according to the manufacturer’s instructions. The sections were imaged using a TS2 inverted microscope.

### Statistical analyses

All the statistical analyses were performed using SPSS software (version 15.0; IBM Corp., Armonk, NY, USA). All experiments were performed at least three times, and the data are expressed as means ± the standard deviation (SD). Data from two groups were compared using an unpaired two-tailed Student’s *t*-test. Data from multiple groups were analyzed using two-way ANOVA with Bonferroni’s multiple comparison test. Differences with *P*-values < 0.05 (*) or < 0.01 (**) were considered statistically significant.

## Results

### E2 promotes the proliferation, migration, and invasion of TNBC cells

**Fig 1:**
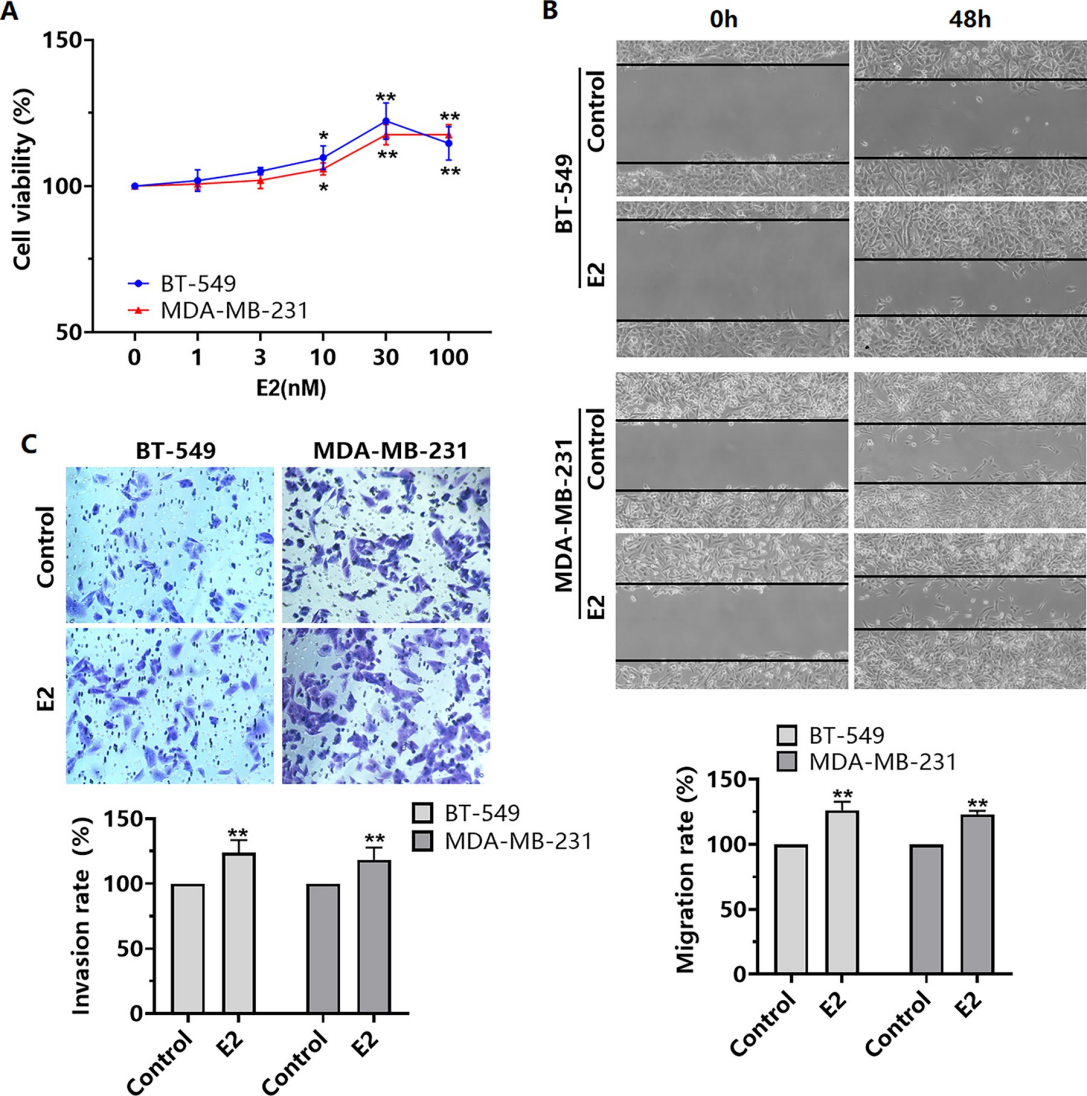
Effect of E2 on TNBC cell proliferation, migration, and invasion. (A) BT-549 and MDA-MB-231 cells were treated with E2 (0–100 nM) for 48 h, and changes in cell viability were then detected by using CCK-8 assay. BT-549 and MDA-MB-231 cells were treated with E2 (30 nM) for 48 h, and (B) cell migration was subsequently assessed by using wound-healing assay and (C) cell invasion was assessed with invasion-chamber assay. *P < 0.05, **P < 0.01 versus the Control.

Our study showed that E2 at 10–100 nM promoted the proliferation of BT-549 and MDA-MB-231 cells, with the greatest effect observed at 30 nM (Fig 1A). Further experiments revealed that 30 nM E2 enhanced the migration and invasion of BT-549 and MDA-MB-231 cells (Fig 1A).

### E2 promotes β-catenin expression and activation in TNBC cells

**Fig 2:**
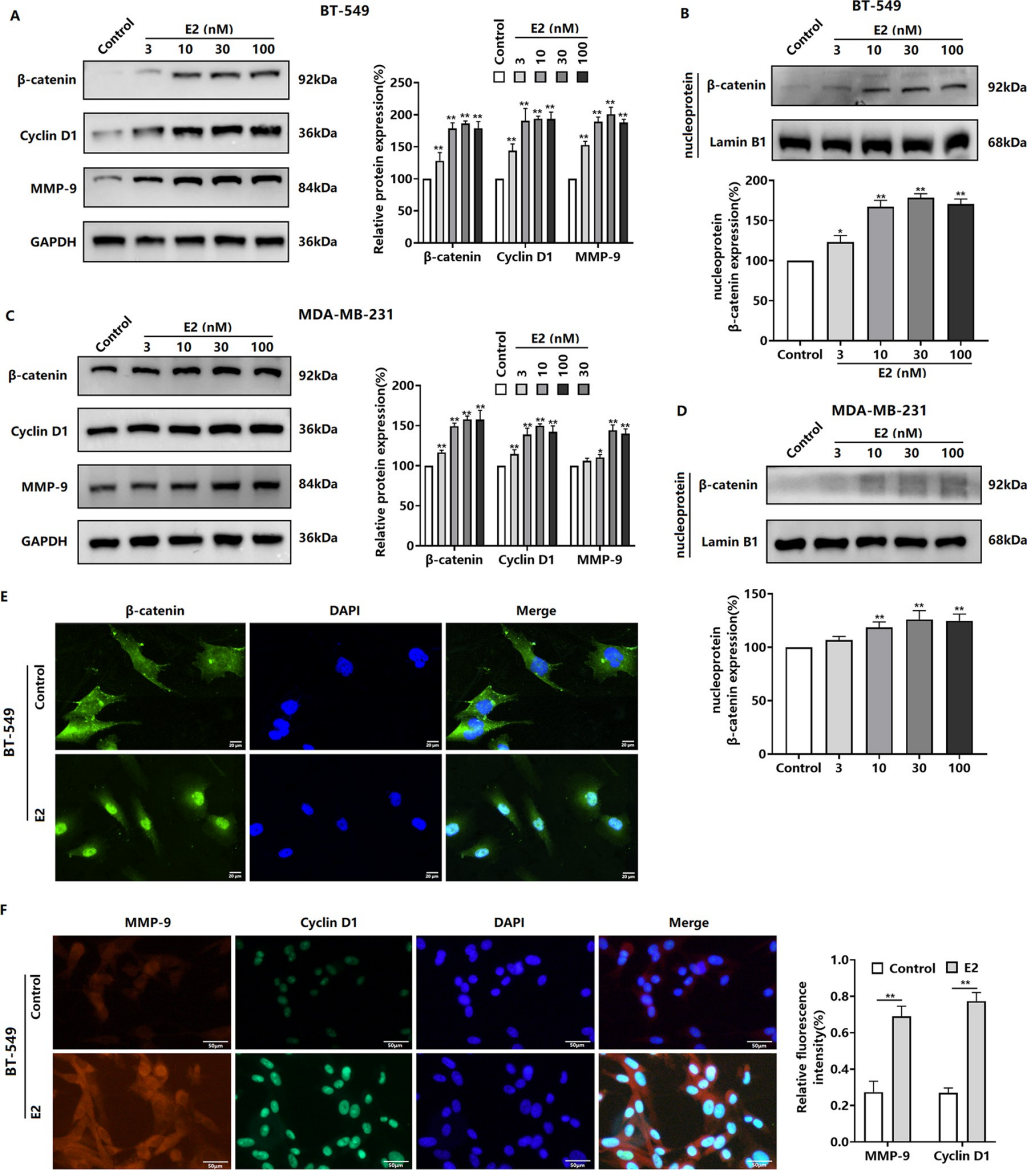
Effect of E2 on β-catenin expression and activity in TNBC cells. (A, C) BT-549 and MDA-MB-231 cells treated with E2 (3–100 nM) 48 h were subjected to western blotting for β-catenin, Cyclin D1, and MMP-9. (B, D) BT-549 and MDA-MB-231 cells treated with E2 (3–100 nM) for 48 h were subjected to western blotting to detect the nuclear levels of β-catenin. BT-549 cells were treated with E2 (30 nM) for 48 h. Immunofluorescence microscopy was performed to detect the subcellular localization of β-catenin, as well as the expression of Cyclin D1 and MMP-9 protein (F). *P < 0.05, **P < 0.01 versus the Control.

β-catenin is an important protein that regulates tumor proliferation, migration, and invasion. We found that E2 upregulated β-catenin protein levels in BT-549 and MDA-MB-231 cells (Fig 2A and 2C). Evaluation of nuclear protein levels revealed that E2 increased the level of β-catenin in the nucleus (Fig 2B and 2D). Then, we examined the effect of E2 on the expression of the β-catenin target genes Cyclin D1 and MMP-9. E2 treatment upregulated Cyclin D1 and MMP-9 protein expression in BT-549 and MDA-MB-231 cells (Fig 2A and 2C). As E2 induced higher expression of β-catenin and its target genes in BT-549 cells than in MDA-MB-231 cells, BT-549 cells were used for the subsequent experiments. Using immunofluorescence microscopy, we found that E2 induced β-catenin nuclear translocation as well as target gene expression in BT-549 cells (Fig 2E and 2F).

### E2 induces the expression and activation of β-catenin in TNBC cells via Calpain

**Fig 3:**
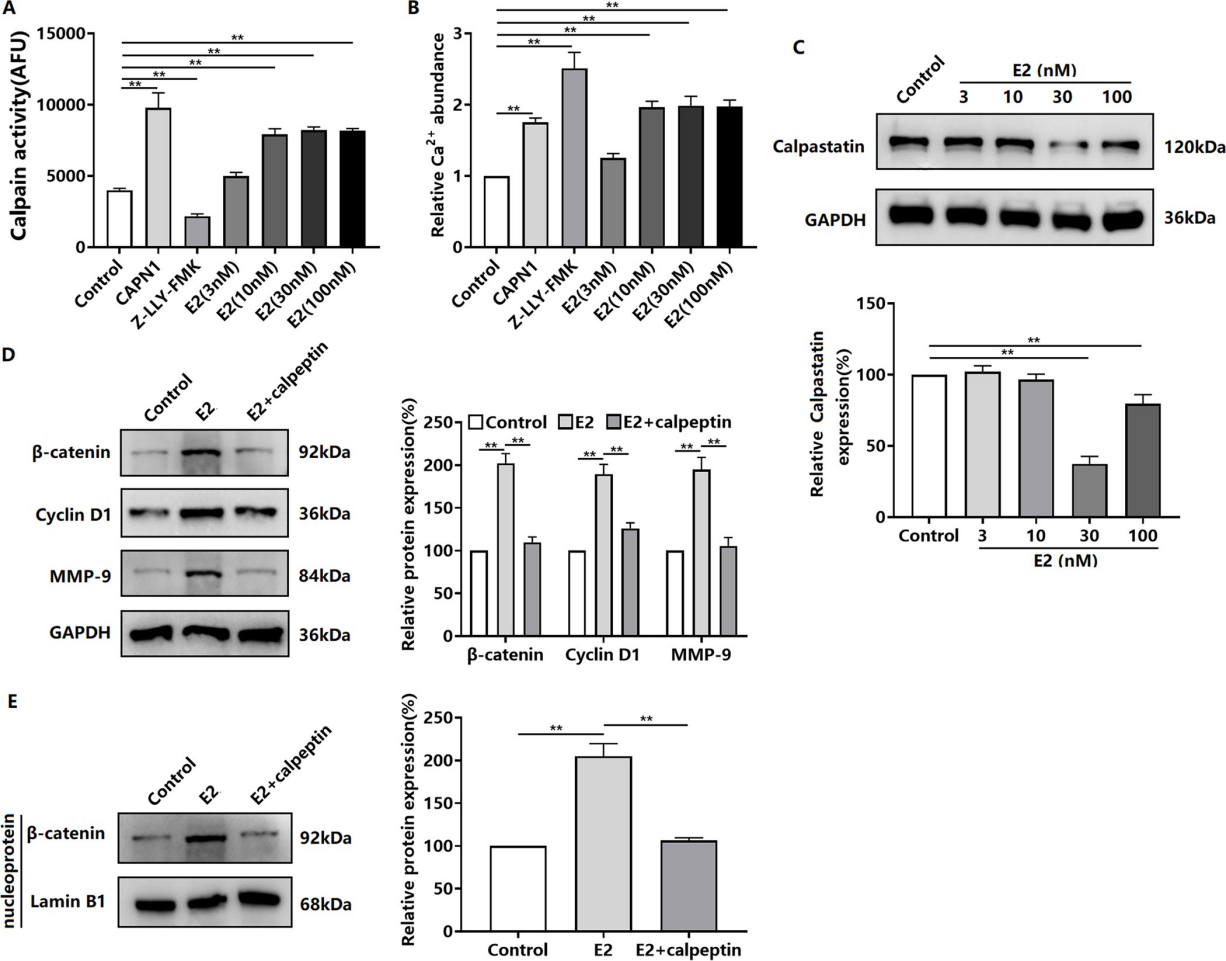
Effect of Calpain on E2-induced β-catenin expression/activation in TNBC cells. BT-549 cells were treated with E2 (3–100 nM) for 48 h. Calpain activity assay (A), determination of Ca^2+^ levels (B), western blot assay of calpastatin expression (C). Western blot assay of the effect of calpeptin on E2-induced β-catenin, Cyclin D1, and MMP-9 protein expression in BT-549 cells (D). Western blot assay of the effect of calpeptin on E2-induced β-catenin protein expression in the nucleus of BT-549 cells (E). **P < 0.01.

We found that E2 treatment increased Calpain activity and Ca^2+^ levels in BT-549 cells (Fig 3A and 3B). Additionally, E2 downregulated the expression of the intracellular Calpain inhibitory protein calpastatin (Fig 3C). To confirm the role of Calpain in E2-induced β-catenin expression and activation in TNBC cells, we investigated the effect of the Calpain inhibitor calpeptin. The results showed that calpeptin inhibited the E2-induced upregulation of β-catenin, Cyclin D1, and MMP-9 protein expression (Fig 3D). Moreover, calpeptin inhibited E2-induced β-catenin accumulation in the nucleus of BT-549 cells (Fig 3E).

### E2 regulates β-catenin through the calpain/YAP signaling axis

**Fig 4:**
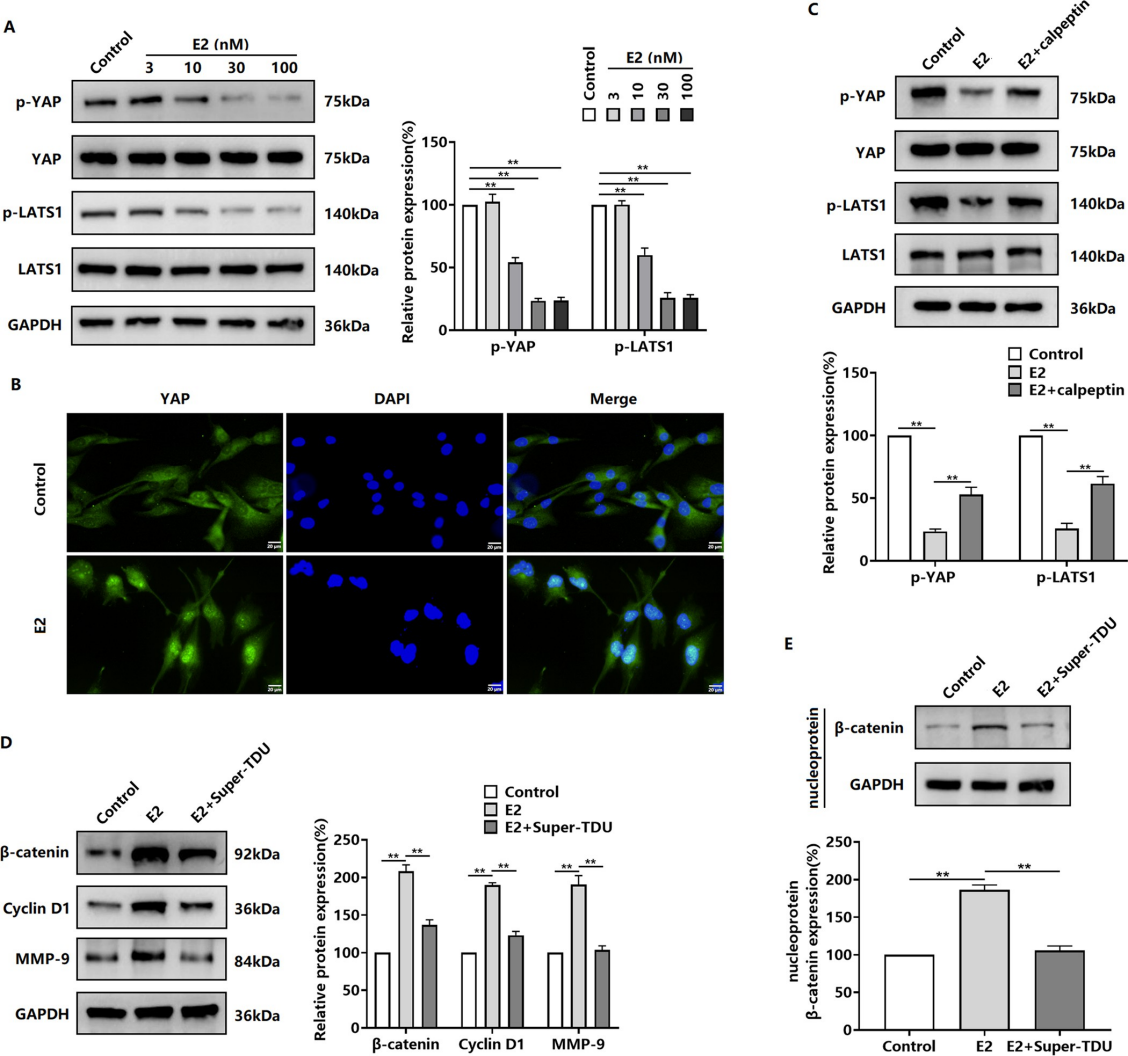
E2 regulation of β-catenin expression and activity is dependent on YAP via Calpain. (A) Western blot detection of phosphorylated LATS1 (p-LATS1) and p-YAP in BT-549 cells treated with 3–100 nM E2 for 48 h. (B) Detection of the effect of E2 on YAP subcellular localization by using immunofluorescence microscopy. (C) Detection of the effect of the Calpain inhibitor calpeptin on E2-induced dephosphorylation of LATS1 and YAP in BT-549 cells by using western blotting. (D) Detection of the effect of the YAP inhibitor Super-TDU on β-catenin, Cyclin D1, and MMP-9 protein expression in E2-induced BT-549 cells via western blotting. (E) Detection of the effect of Super-TDU on β-catenin levels in the nuclei of E2-induced BT-549 cells by using western blotting. **P < 0.01.

Further studies revealed that E2 induced the dephosphorylation of YAP, a core molecule in the Hippo signaling pathway, and its entry into the nucleus by inhibiting the phosphorylation of LATS1 (Fig 4A and 4B). Calpeptin pretreatment inhibited the effect of E2 on YAP dephosphorylation (Fig 4C), suggesting that calpain mediated the E2-induced translocation of YAP into the nucleus of BT-549 cells. To verify that calpain regulates β-catenin expression and activation through YAP, we inhibited YAP transcriptional activity with Super-TDU and then observed the inducing effect of E2. The results showed that Super-TDU inhibited the E2-induced upregulation of β-catenin, Cyclin D1, and MMP-9 protein expression (Fig 4D). In addition, Super-TDU inhibited the E2-induced accumulation of β-catenin in the nucleus of BT-549 cells (Fig 4E).

### E2 regulates the proliferation, migration, and invasion of TNBC cells via the Calpain/YAP/β-catenin axis

**Fig 5:**
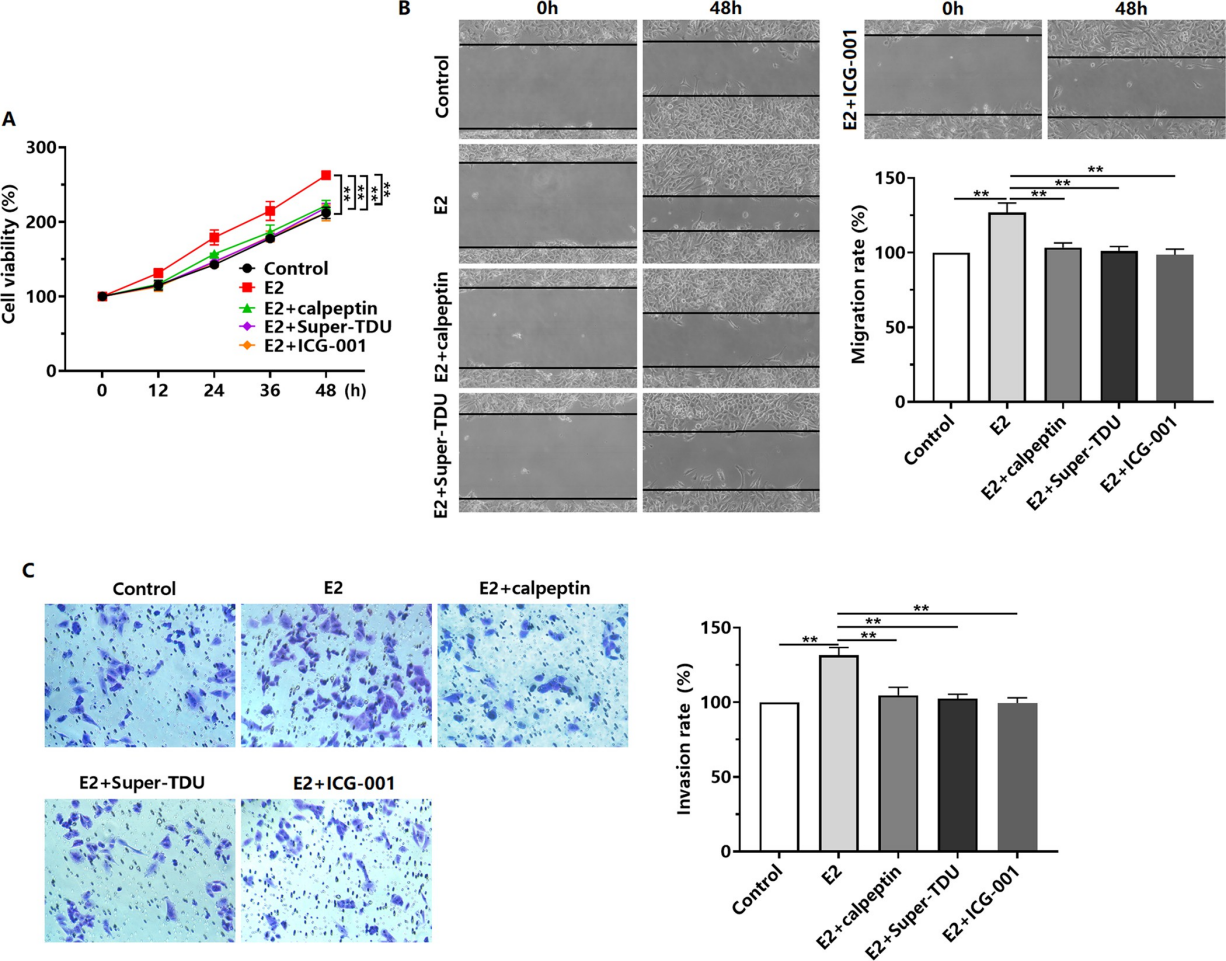
Effects of inhibiting the Calpain/YAP/β-catenin pathway on E2-induced TNBC cell proliferation, migration, and invasion. (A–C) Effects of calpeptin (10 μM, Calpain inhibitor), Super-TDU (10 μM, YAP inhibitor), or ICG-001 (5 μM, β-catenin inhibitor) pretreatment for 4 h on the (A) proliferation, (B) migration (wound-healing assay), and (C) invasion (invasion-chamber assay) of BT-549 cells treated with E2 for 48 h. **P < 0.01.

We observed the effects of inhibiting Calpain, YAP, or β-catenin on E2-induced proliferation, migration, and invasion of BT-549 cells. The results showed that pretreatment with calpeptin, Super-TDU, or ICG-001 inhibited the E2-induced proliferation, migration, and invasion of BT-549 cells (Fig 5A~5C).

### E2 promotes metastasis of TNBC through the Calpain/β-catenin signaling axis

**Fig 6:**
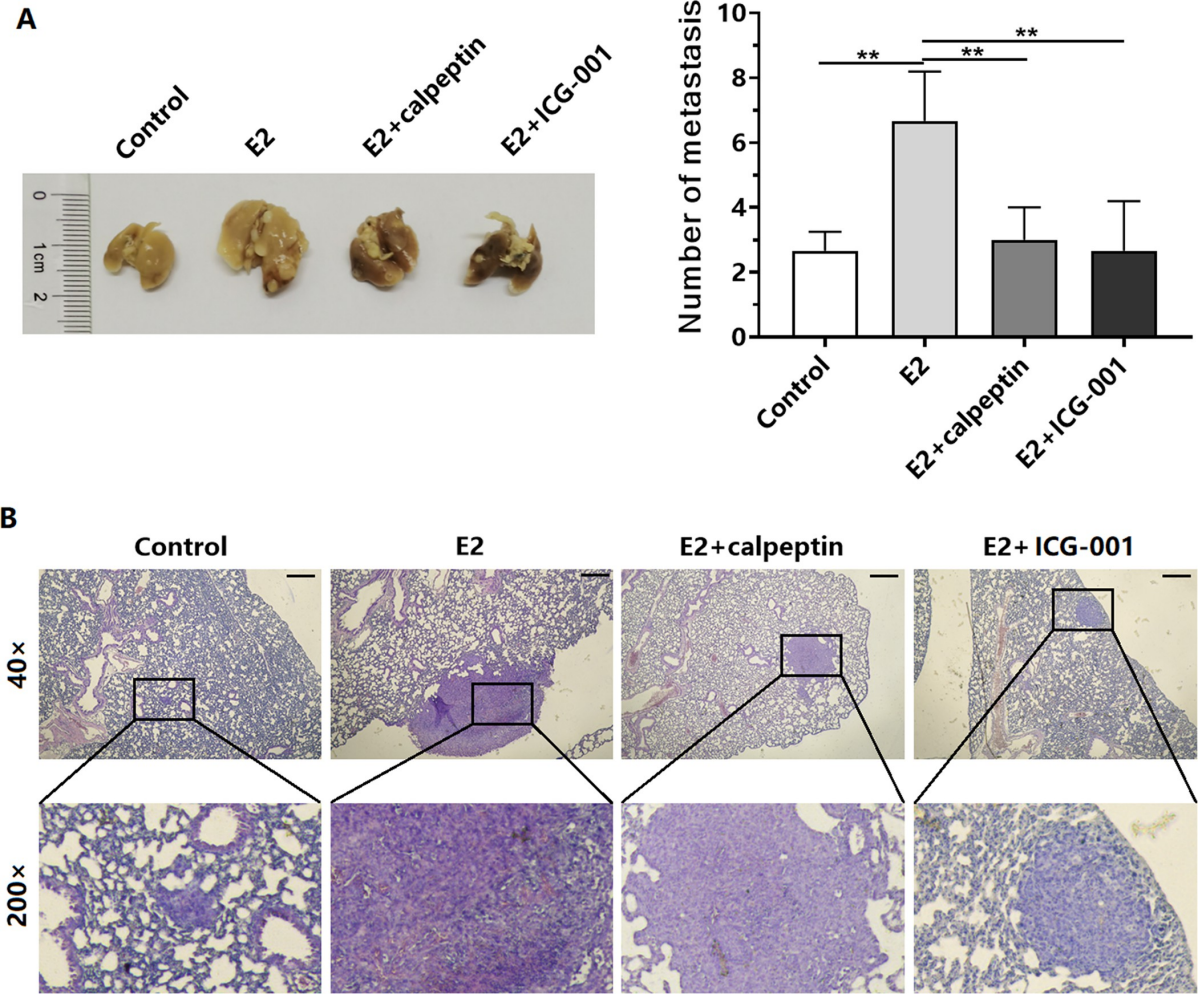
E2 promotes metastasis of TNBC cells in mice via the Calpain/β-catenin signaling axis. (A) Representative graphs of lung metastatic foci and statistical analysis of the number of metastatic foci in the Control, E2, E2 + calpeptin, and E2 + ICG-001 groups. (B) Representative graphs of H&E-stained lung metastases in mice from each group. Scale bar, 200 μm. **P < 0.01.

We investigated the effect of E2 on BT549 cell metastasis and explored the role of the Calpain/β-catenin signaling axis in the observed effects by using a mouse model. The results showed that E2 induced lung metastasis of BT-549 cells injected into NOD-SCID mice and that treatment with calpeptin or ICG-001 inhibited the E2-induced lung metastasis (Fig 6A and 6B).

## Discussion

E2 is considered to induce breast cancer cell proliferation through the classical ER signaling pathway. However, in ER-deficient TNBC, E2 promotes malignant tumor progression. Studies have shown that E2 can promote cell viability and motility through non-genomic effects induced by G protein-coupled estrogen receptors [18]. In addition, E2 induces brain metastasis of TNBC cells through the activation of tumor cell tropomyosin kinase receptor B via brain-derived neurotrophic factor [10]. Consistent with previous reports, our study shows that E2 induces the proliferation, migration, invasion, and metastasis of BT-549 and MDA-MB-231 TNBC cells. Furthermore, we found that E2 increased β-catenin protein expression. Moreover, E2 induced the translocation of β-catenin into the nucleus, where it promoted the expression of the target genes Cyclin D1 and MMP-9. The inhibition of β-catenin blocked these E2-induced malignant behaviors. Studies have reported that β-catenin mediates TNBC cell migration and invasion by activating kinesin family member 23 (KIF23) [19]. β-Catenin also mediates integrin α9-induced activation of TNBC cell proliferation and metastasis [20]. Our results suggest that E2 induced the metastasis of BT-549 TNBC cells via β-catenin signaling. Studies have shown that E2 can induce β-catenin activation via the ER and lymphoid enhancer-binding factor-1 [21]. However, the mechanism by which E2 induces β-catenin activation in ER-deficient TNBC requires further investigation.

Calpain is a calcium-activated cysteine protease that catalyzes the hydrolysis of specific substrates [22–24]. Calpain activity is regulated by intracellular Ca^2+^ levels, the Calpain inhibitor calpastatin, and Calpain activators [22–24]. Calpain substrates are involved in various cellular processes, including transcription, survival, apoptosis, migration, and angiogenesis [22–24]. Dysregulation of Calpain activity is closely associated with the development of various tumors, including breast cancer. The Calpain family member calpain-1 is expressed in TNBC and associated with prognosis [25]. Calpain has been reported to mediate fibronectin-induced epithelial–mesenchymal transition in breast cancer cells [26]. E2 enhances the adhesion of breast cancer cells via the G protein-coupled estrogen receptor–-Calpain signaling axis. In the present study, E2 enhanced Calpain activity by increasing intracellular Ca^2+^ levels and downregulating calpastatin expression. Inhibition of Calpain reversed the effects of E2 on the expression and nuclear translocation of β-catenin. In addition, inhibition of Calpain blocked E2-induced cell proliferation, migration, invasion, and metastasis, suggesting that E2 induced the metastasis of BT-549 TNBC cells by activating the Calpain/β-catenin signaling axis. However, the mechanism by which Calpain activates β-catenin remains unclear.

**Fig 7:**
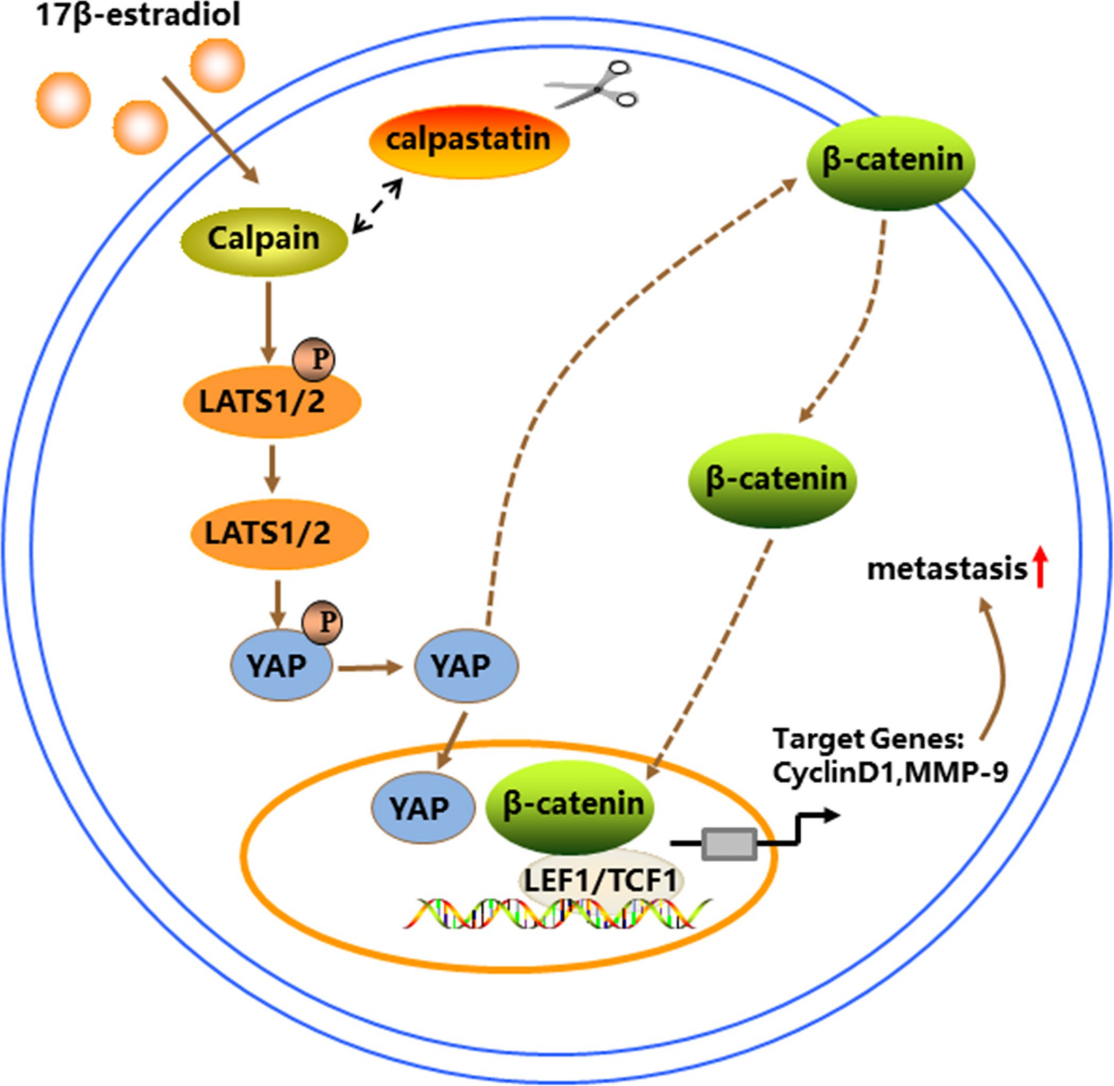
E2 induces TNBC cell metastasis through the Calpain/YAP/β-catenin signaling axis. E2 activates Calpain by downregulating calpastatin expression and increasing Ca^2+^ levels. Activated Calpain induces YAP translocation into the nucleus by dephosphorylating LATS1/2 and YAP via the LATS1/2-YAP signaling axis. YAP enters the nucleus and regulates β-catenin expression and entry into the nucleus while also promoting the expression of the target genes Cyclin D1 and MMP-9, thereby promoting the metastasis of TNBC cells.

Our study also revealed that E2 treatment resulted in the dephosphorylation and translocation of YAP into the nucleus in BT-549 cells. YAP is a transcriptional coactivator downstream of Hippo signaling, and it is involved in various processes related to tumor development [27, 28]. Calpain-6, a Calpain family member, has been reported to promote the proliferation and metastasis of sarcoma stem cells by activating YAP [29]. Therefore, we hypothesized that YAP acts downstream of Calpain upon induction of BT-549 cells by E2. Further experiments revealed that Calpain inhibition reversed the effects of E2 on BT-549 cell proliferation, migration, and invasion, as well as YAP dephosphorylation. Therefore, E2 may promote the malignant behavior of BT-549 cells through the Calpain/YAP signaling axis. Studies have shown that YAP activates the Wnt/β-catenin signaling pathway in intestinal cells to promote self-renewal and tumorigenesis [30]. The YAP/β-catenin signaling pathway also mediates the effects of M2 macrophage-derived extracellular vesicles on CD8^+^ T cell exhaustion in hepatocellular carcinoma [31]. YAP has been reported to promote glioma cell proliferation by regulating the expression, subcellular localization, and transcriptional activity of β-catenin through GSK3β [32]. This suggests that YAP is an upstream signaling target of β-catenin. We found that the inhibition of YAP transcriptional activity reversed the β-catenin-inducing effects of E2. Simultaneous inhibition of YAP reversed the E2-mediated induction of BT-549 cell proliferation, migration, invasion, and metastasis. All these results indicate that the YAP/β-catenin signaling pathway mediated the effects of E2 on BT-549 cells. These results suggest that E2 regulates TNBC metastasis through the Calpain/YAP/β-catenin signaling pathway (Fig 7).

## Conclusion

Our results suggest the presence of a functional Calpain/YAP/β-catenin signaling pathway in TNBC cells that mediates E2-induced metastasis. Our study results revealed a potential mechanism by which E2 induces TNBC metastasis, and that disruption of the Calpain/YAP/β-catenin signaling pathway may be a promising target to block the pro-cancer effects of E2.

## Supporting information

S1 Raw images (PDF)

S1 Data The minimum data. (DOCX)
