# Supplementary figures and images for: 17β-Estradiol promotes metastasis in triple-negative breast cancer through the Calpain/YAP/β-catenin signaling axis
Source: PLoS One. 2024 Mar 28;19(3):e0298184. doi: 10.1371/journal.pone.0298184 (PMC10977805; doi:10.1371/journal.pone.0298184)

**Fig. 2A BT-549:**

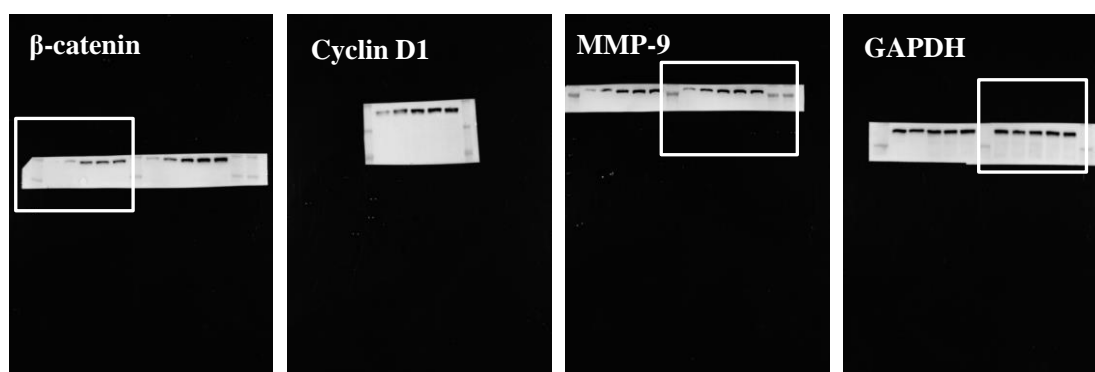

**Fig. 2B BT-549:**

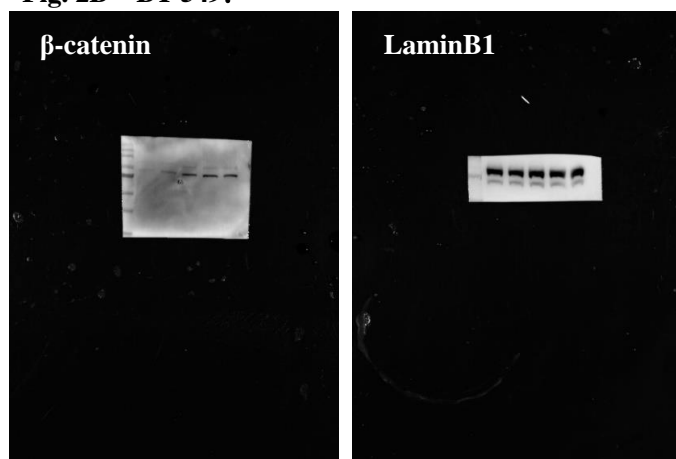

**Fig. 2C MDA-MB-231:**

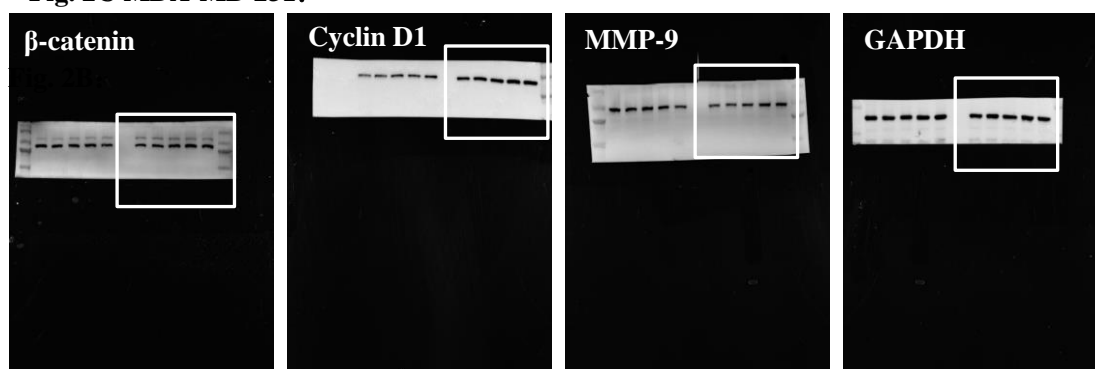

**Fig. 2D MDA-MB-231:**

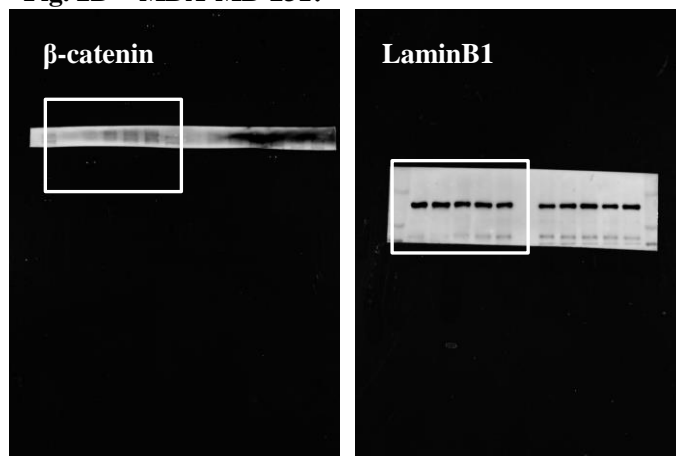

**Fig. 3C:**

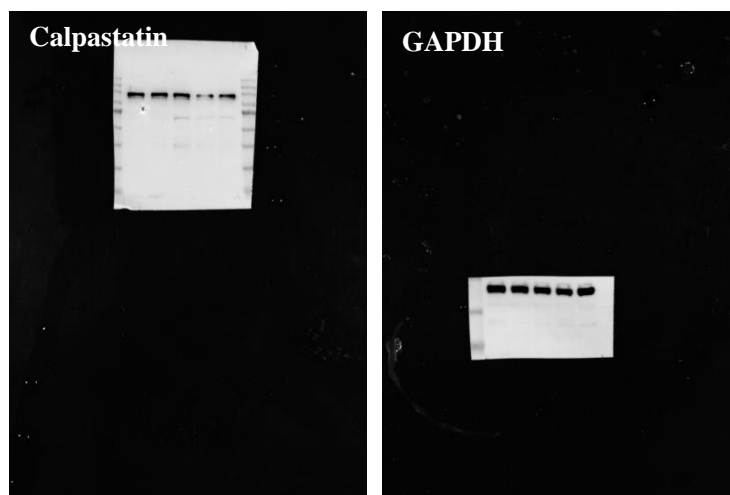

**Fig. 3D:**

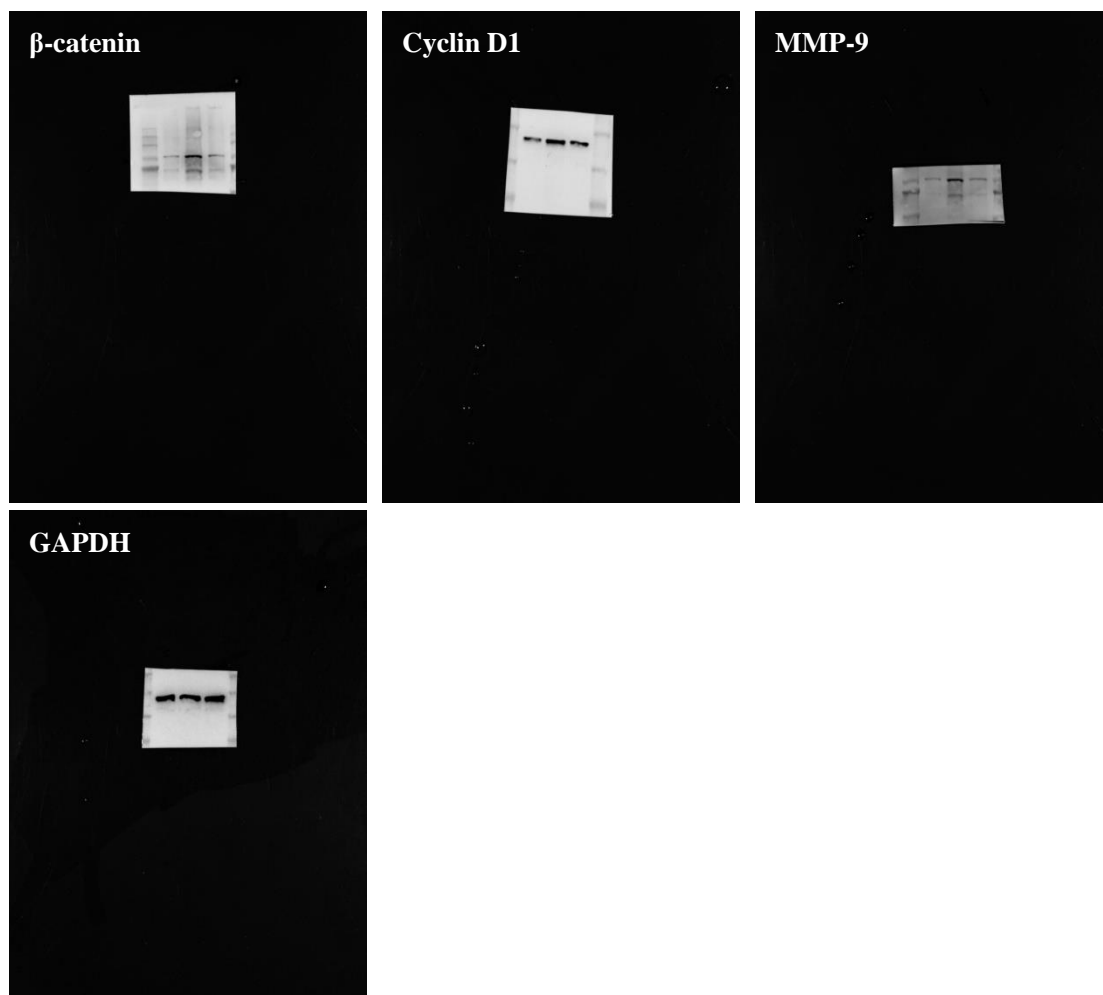

**Fig. 3E:**

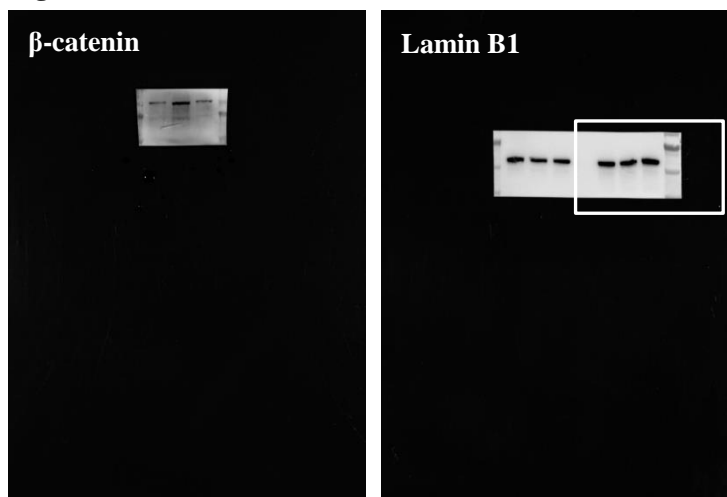

**Fig. 4A:**

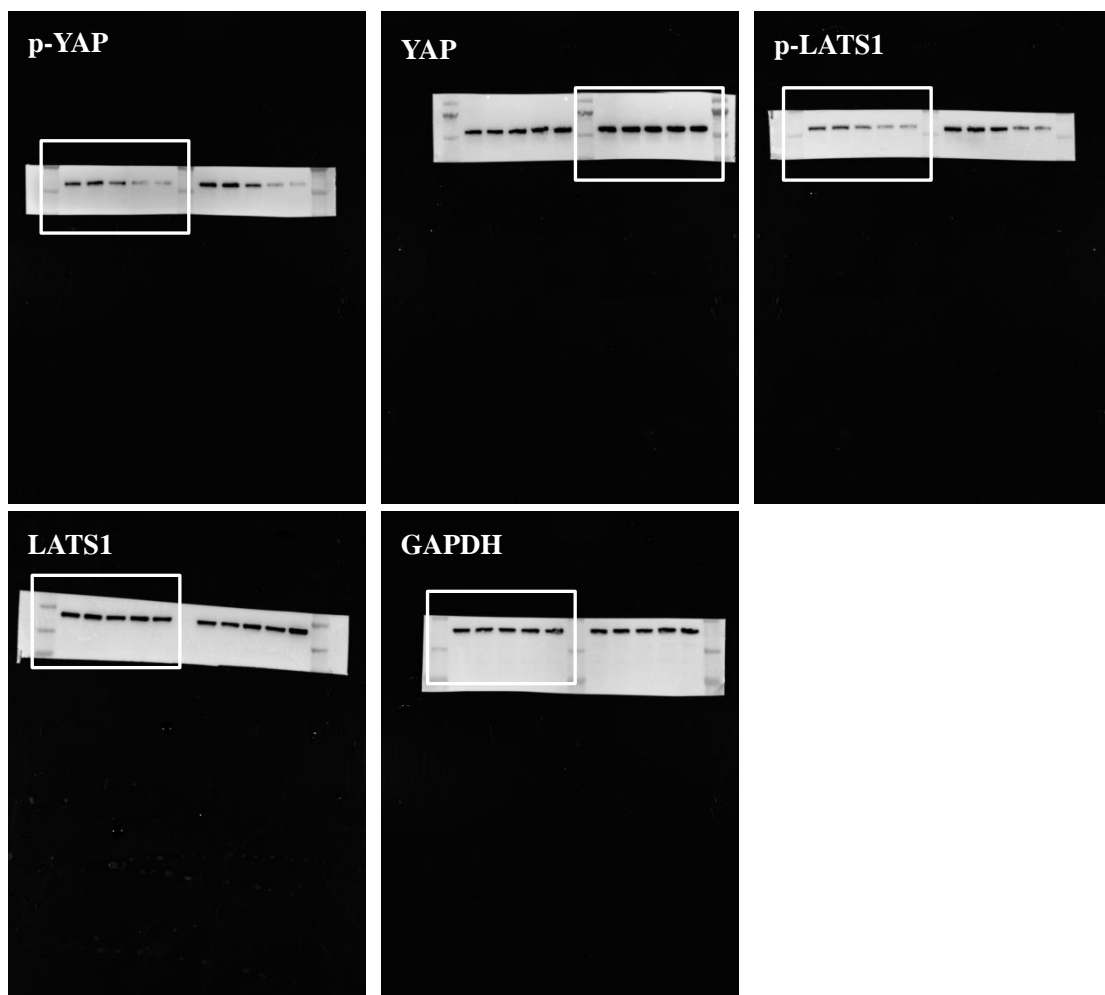

**Fig. 4C:**

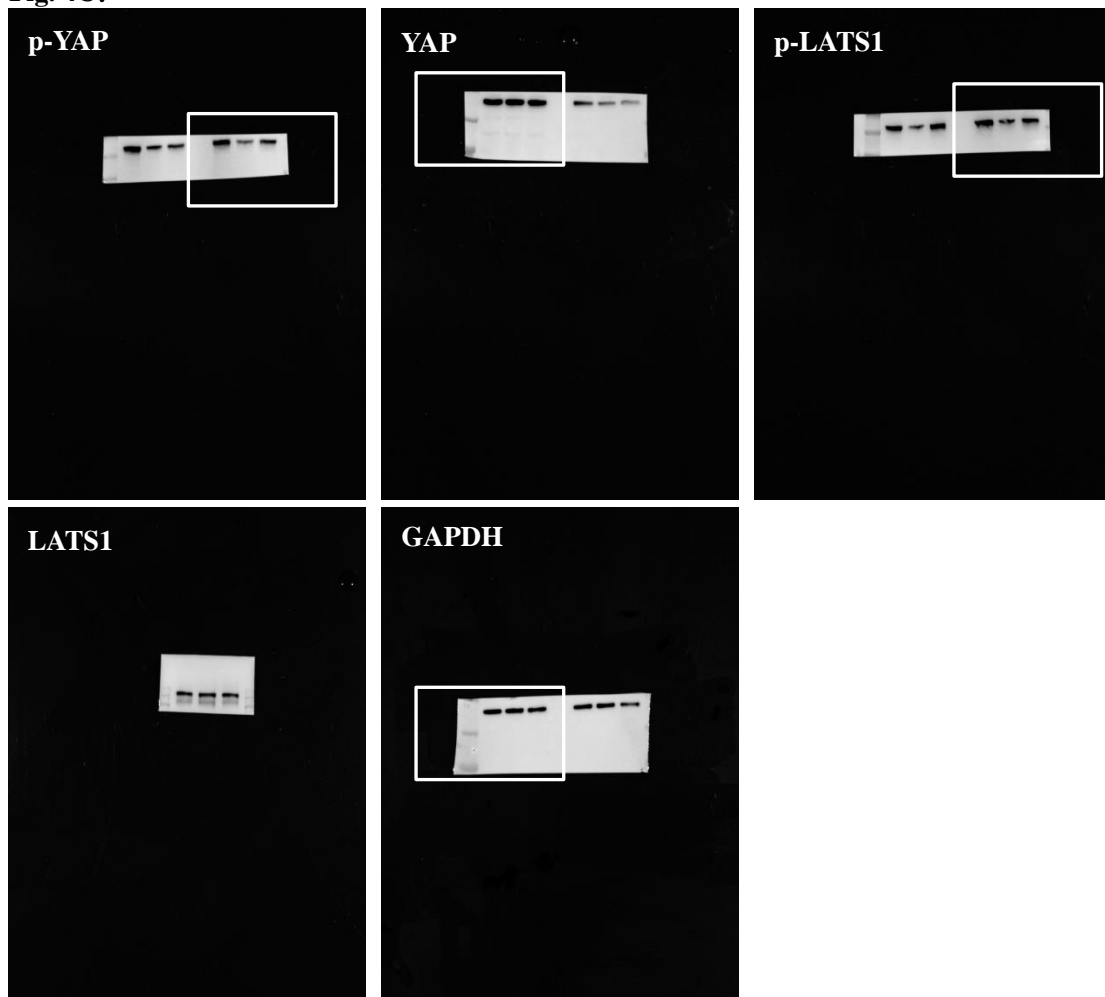

**Fig. 4D:**

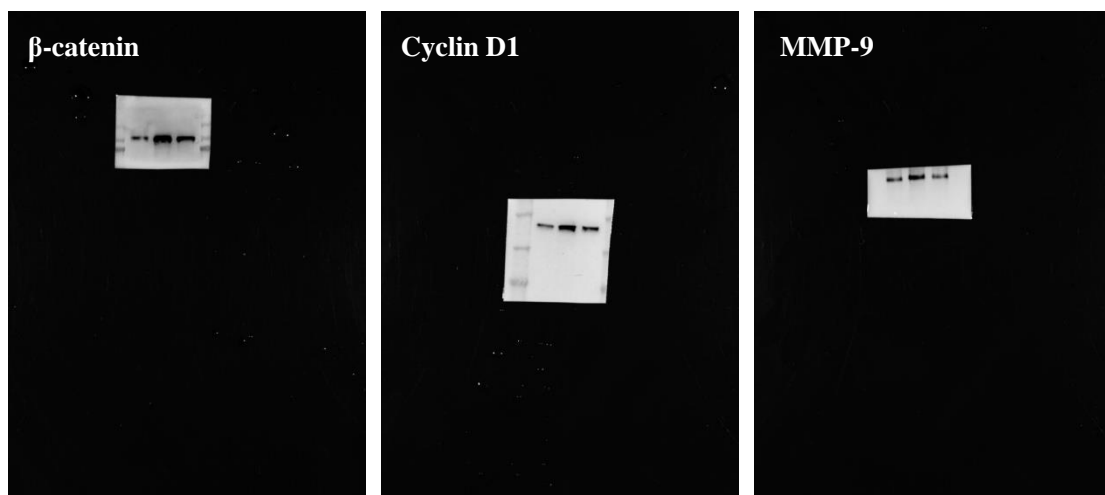

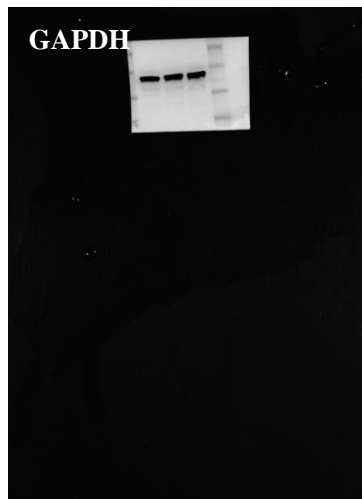

**Fig. 4E:**

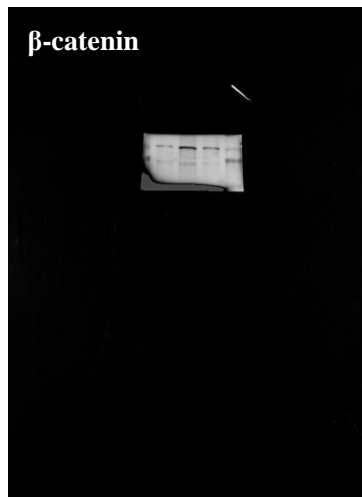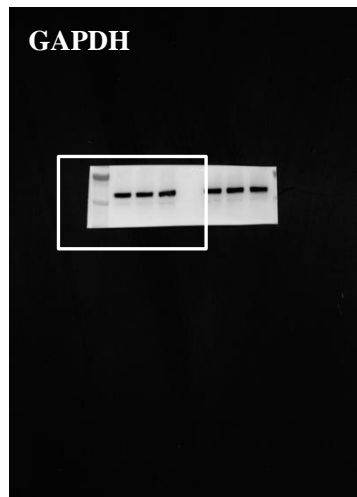

Supplement: S1 Raw images — (PDF) [file pone.0298184.s001.pdf]
