# Supplementary material for: 17β-Estradiol promotes metastasis in triple-negative breast cancer through the Calpain/YAP/β-catenin signaling axis
Source: PLoS One. 2024 Mar 28;19(3):e0298184. doi: 10.1371/journal.pone.0298184 (PMC10977805; doi:10.1371/journal.pone.0298184)
Supplement: S1 Data — (DOCX) [file pone.0298184.s002.docx]

**Fig. 1A**

| **E2（nM）** | BT-549 | | | MDA-MB-231 | | |
| --- | --- | --- | --- | --- | --- | --- |
| **0** | 100 | 100 | 100 | 100 | 100 | 100 |
| **1** | 98.6 | 105.8 | 101.2 | 100.3 | 102.6 | 99.1 |
| **3** | 103.8 | 106.2 | 105.3 | 99.7 | 105.2 | 101.1 |
| **10** | 110.7 | 105.3 | 113.2 | 103.5 | 107.3 | 106.8 |
| **30** | 121.3 | 116.5 | 128.9 | 114.3 | 117.2 | 121.2 |
| **100** | 116.3 | 108.2 | 119.3 | 113.5 | 118.6 | 120.9 |

**Fig. 1B**

**BT-549 MDA-MB-231**

**
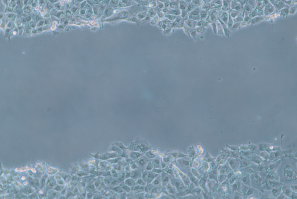

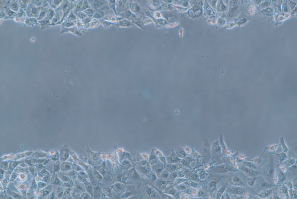

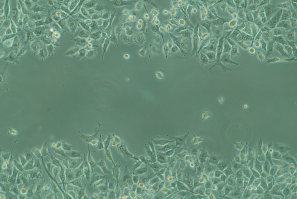

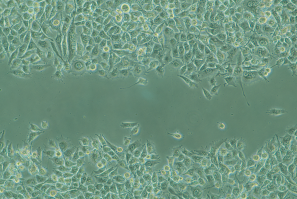
**

**
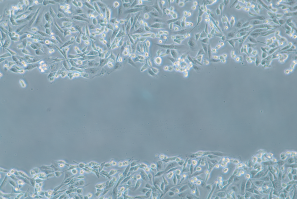

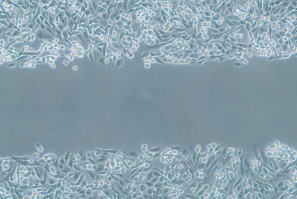

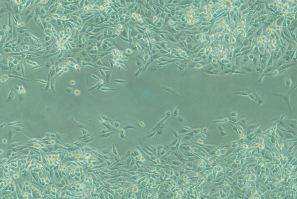

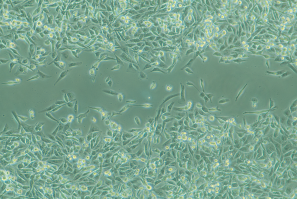
**

**
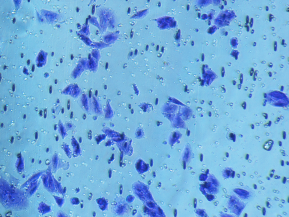

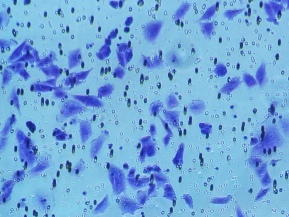

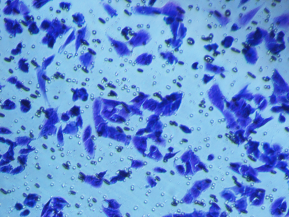

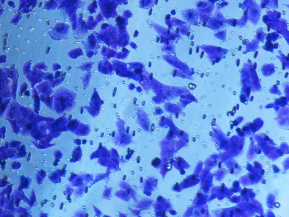
**

**
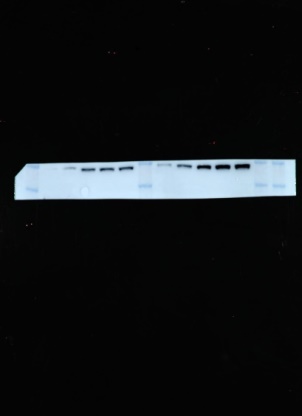

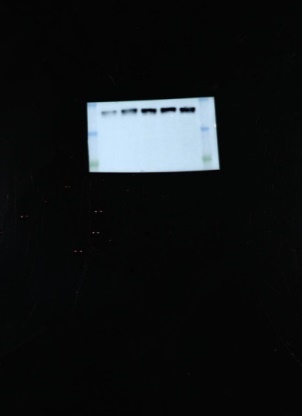

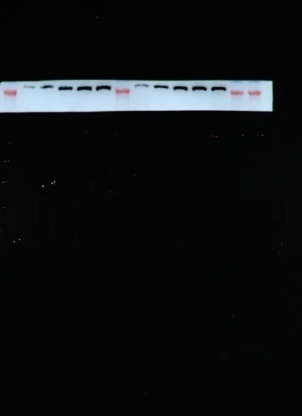

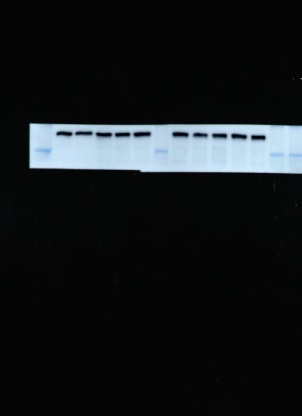
**

**Fig. 2B BT-549：**

**GAPDH**

**MMP-9**

**Cyclin D1**

**β-catenin**

**Fig. 2A BT-549：**

**
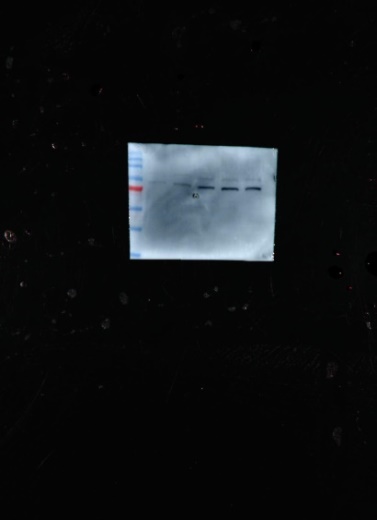

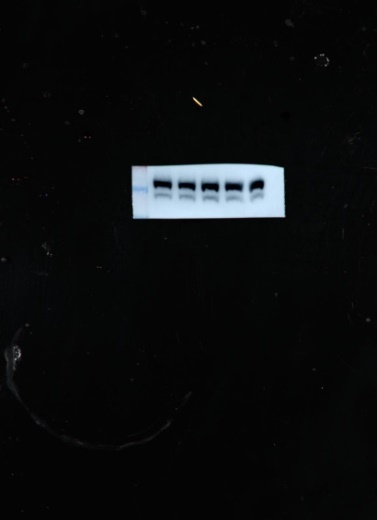
**

**Fig. 2C MDA-MB-231：**

**β-catenin**

**LaminB1**

**GAPDH**

**Cyclin D1**

**
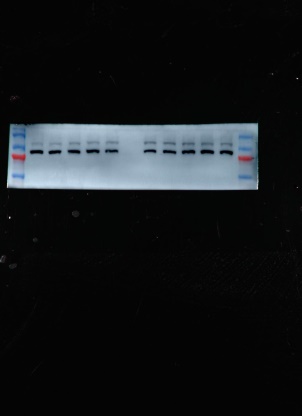

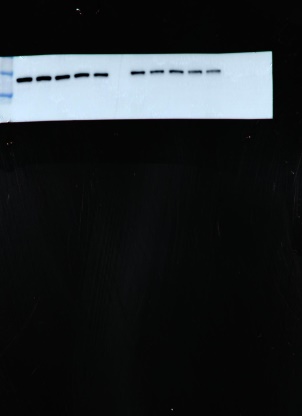

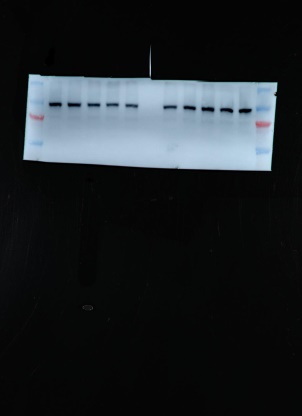

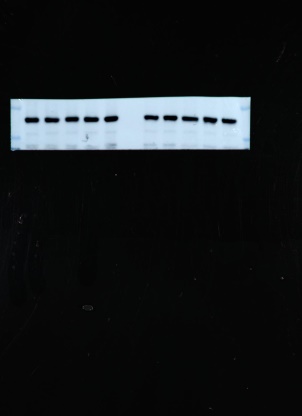
**

**Fig. 2D MDA-MB-231：**

**β-catenin**

**MMP-9**

**Fig. 2B：**


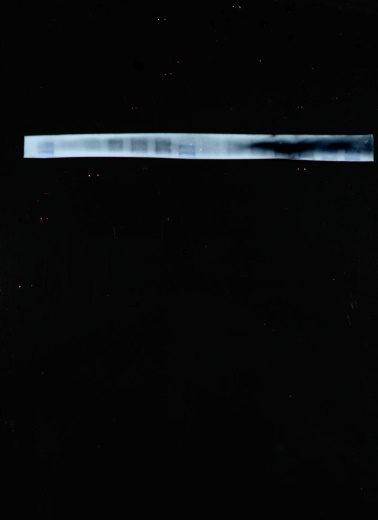

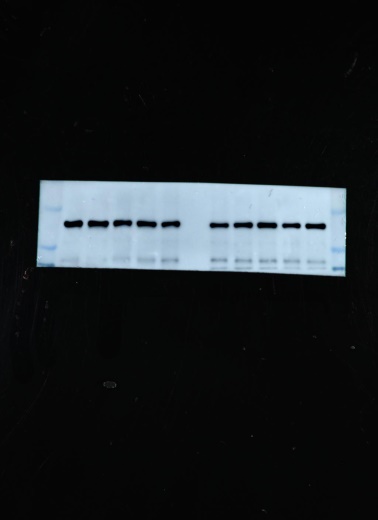


**Fig. 2E BT-549：**

**LaminB1**

**β-catenin**


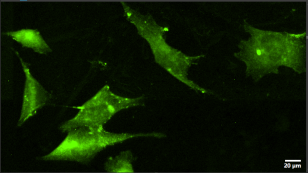

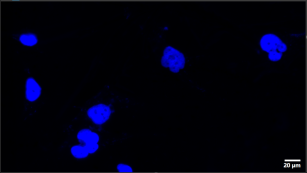

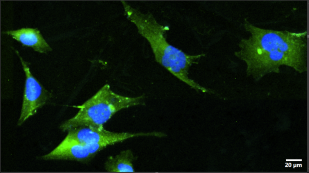


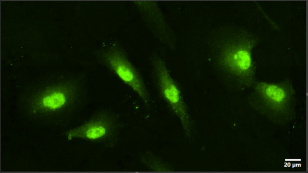

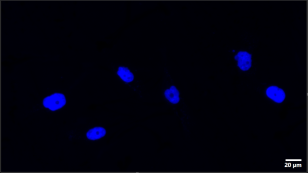

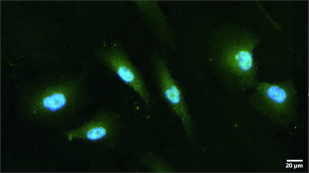


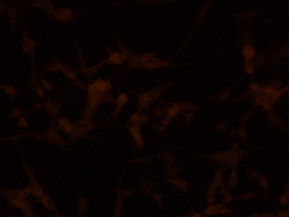

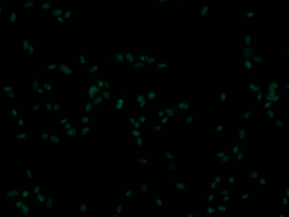

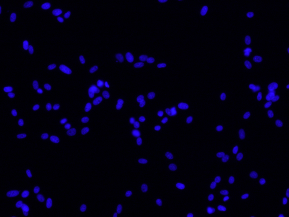

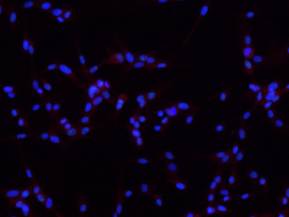

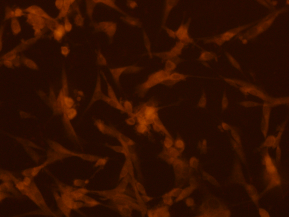

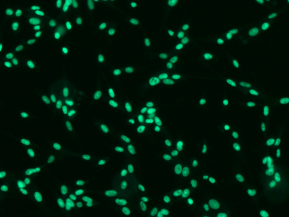

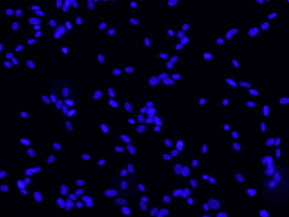

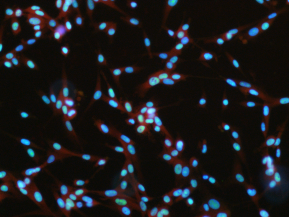


**Fig. 3A**

**Fig. 2F BT-549：**

| Control | | | CAPN1 | | | Z-LLY-FMK | | | E2(3nM) | | | E2(10nM) | | | E2(30nM) | | | E2(100nM) | | |
| --- | --- | --- | --- | --- | --- | --- | --- | --- | --- | --- | --- | --- | --- | --- | --- | --- | --- | --- | --- | --- |
| 3856  **Fig. 3B** | 4011 | 4125 | 10879 | 8768 | 9658 | 2178 | 2336 | 1992 | 5025 | 4718 | 5233 | 7876 | 8325 | 7538 | 8223 | 7976 | 8425 | 8017 | 8119 | 8339 |

| Control | | | CAPN1 | | | Z-LLY-FMK | | | E2(3nM) | | | E2(10nM) | | | E2(30nM) | | | E2(100nM) | | |
| --- | --- | --- | --- | --- | --- | --- | --- | --- | --- | --- | --- | --- | --- | --- | --- | --- | --- | --- | --- | --- |
| 1 | 1 | 1 | 1.73 | 1.82 | 1.7 | 2.26 | 2.58 | 2.69 | 1.26 | 1.19 | 1.31 | 1.87 | 1.99 | 2.03 | 1.98 | 2.12 | 1.86 | 1.89 | 2.07 | 1.96 |


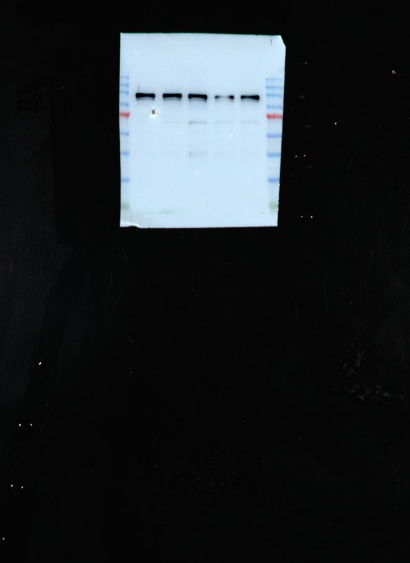

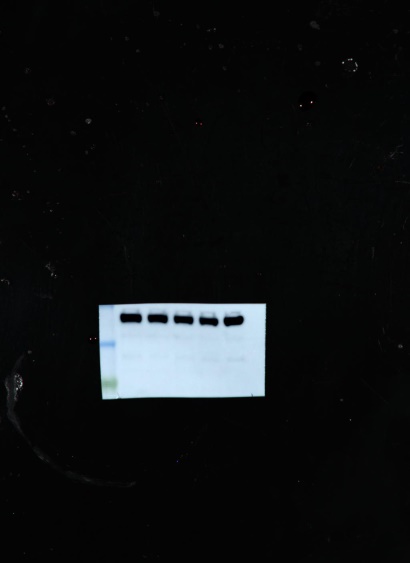


**Fig. 3D：**

**GAPDH**

**Calpastatin**

**Fig. 3C：**


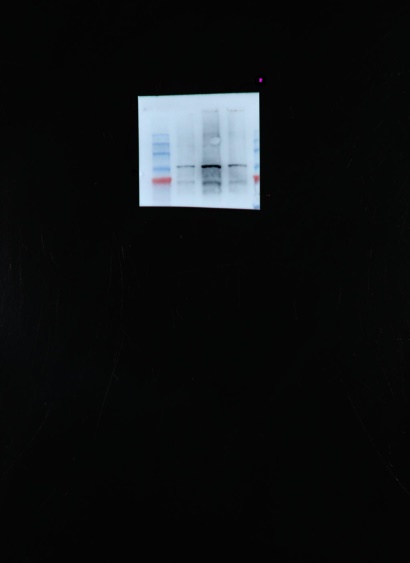

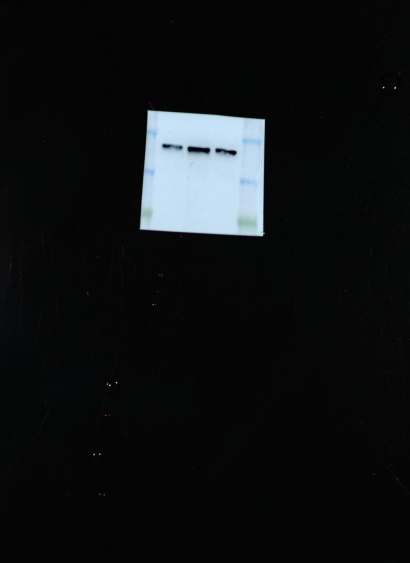

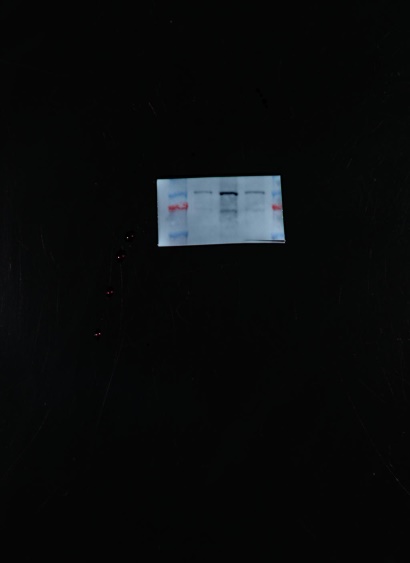


**MMP-9**

**Cyclin D1**

**β-catenin**


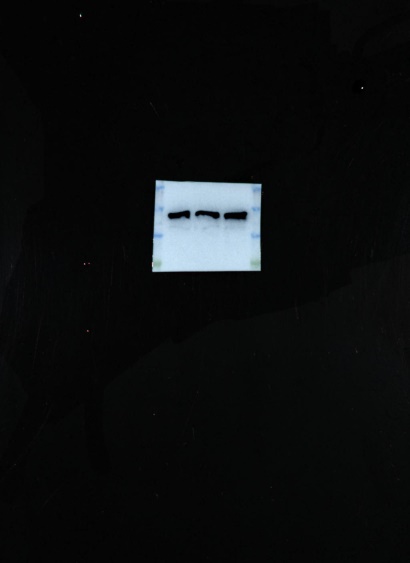


**GAPDH**


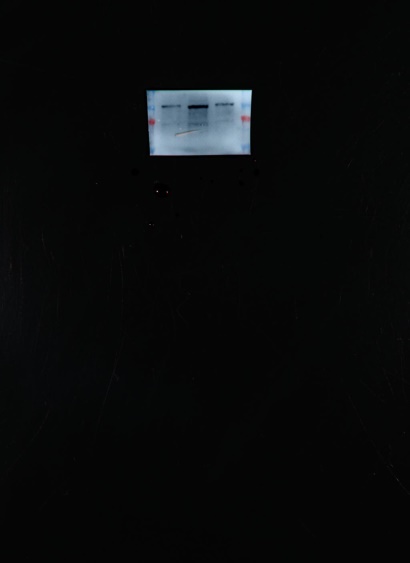

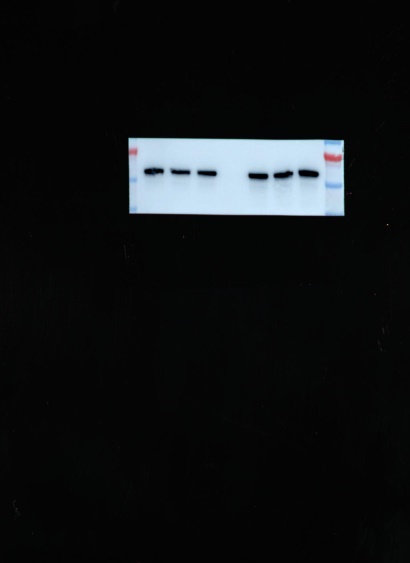


**Lamin B1**

**β-catenin**

**Fig. 3E：**

**Fig. 4A：**


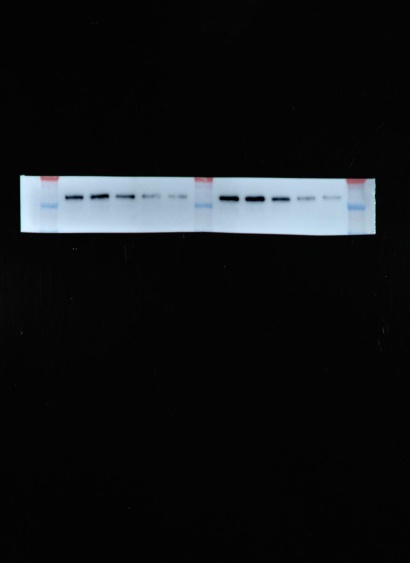

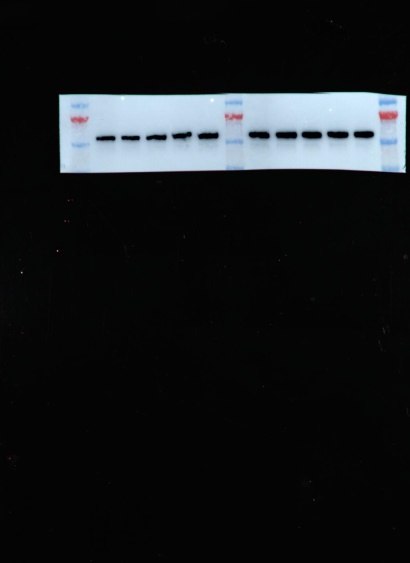

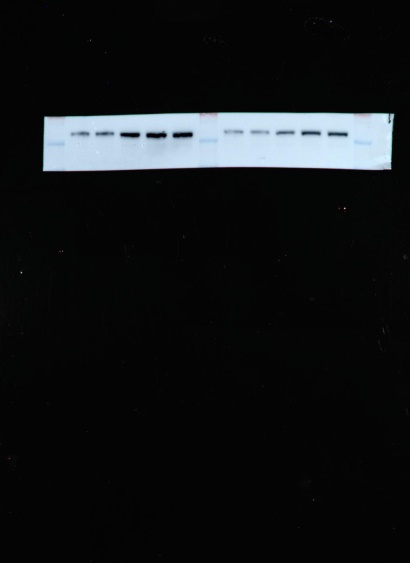


**p-LATS1**

**YAP**

**p-YAP**


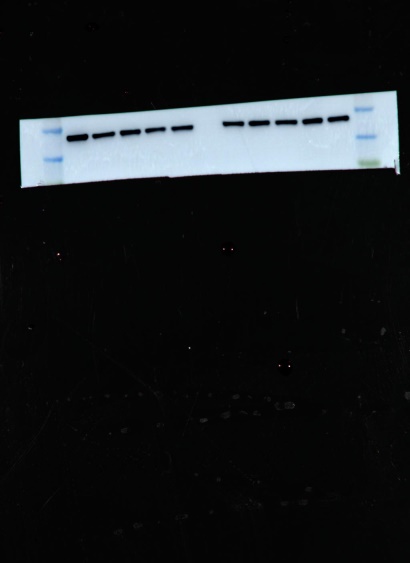

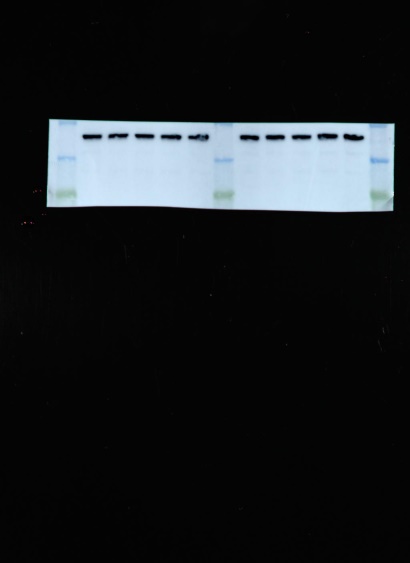


**GAPDH**

**LATS1**

**Fig. 4B：**


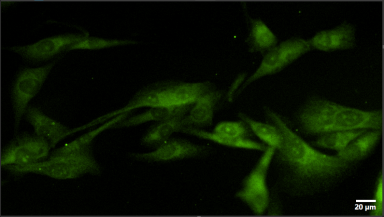

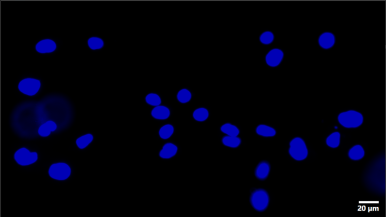

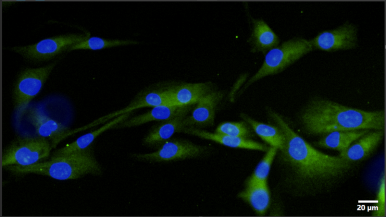

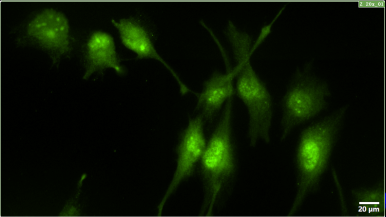

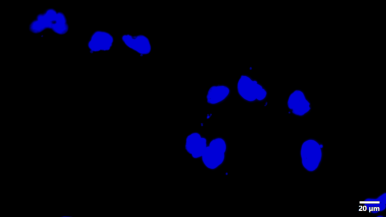

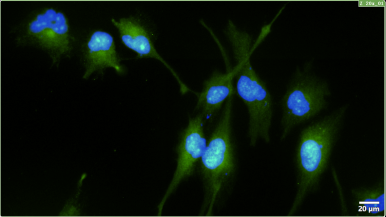


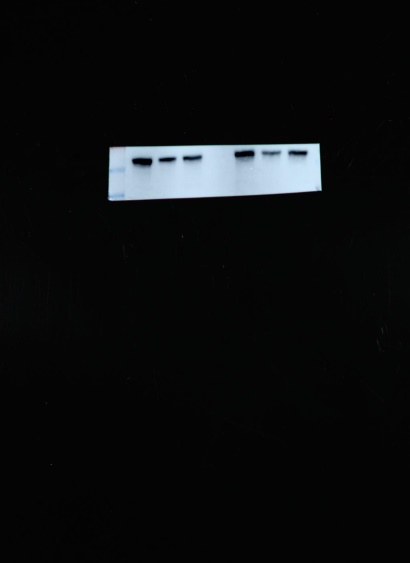

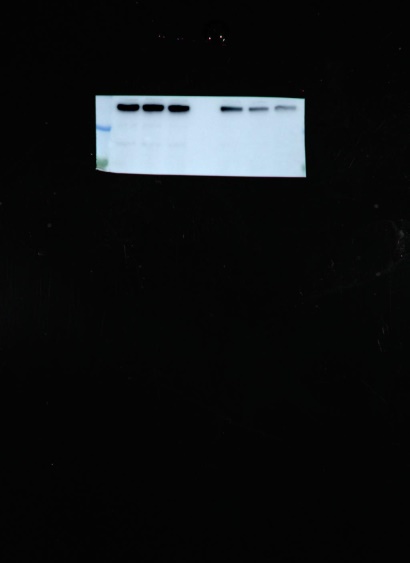

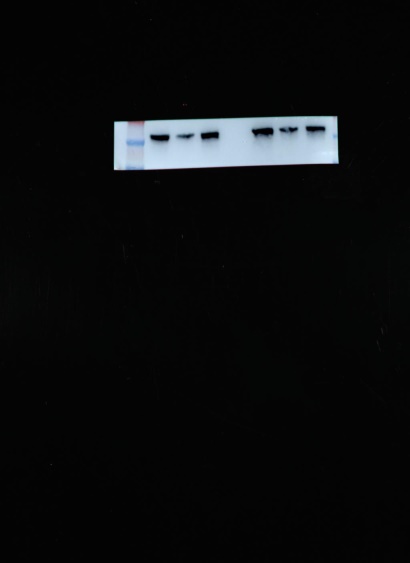


**p-LATS1**

**YAP**

**p-YAP**

**Fig. 4C：**


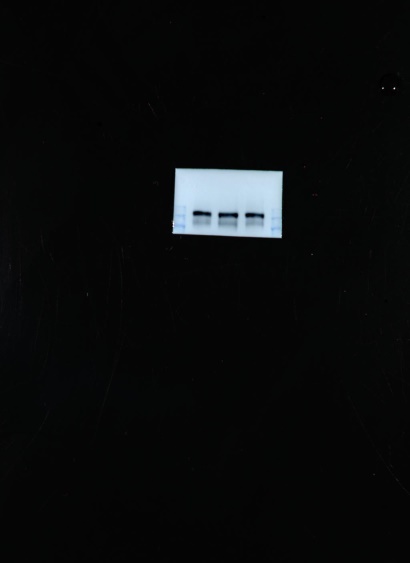

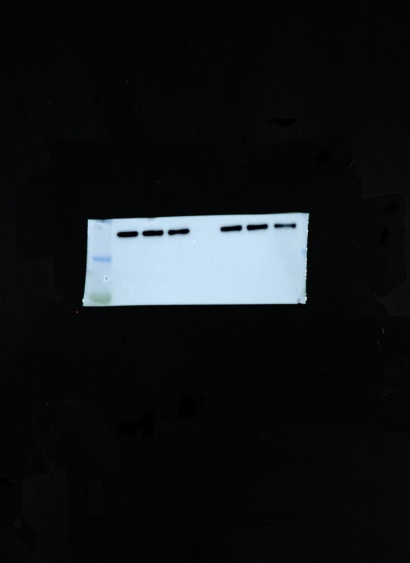


**Fig. 4D：**

**GAPDH**

**LATS1**


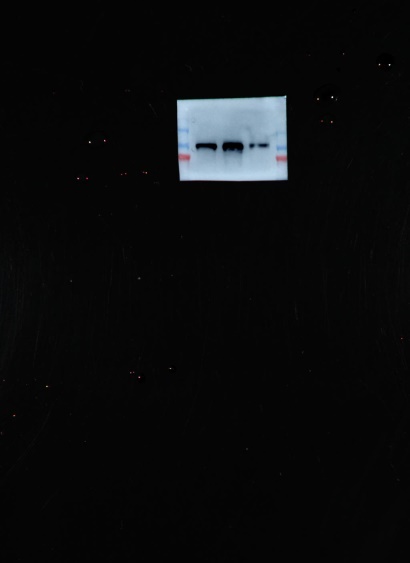

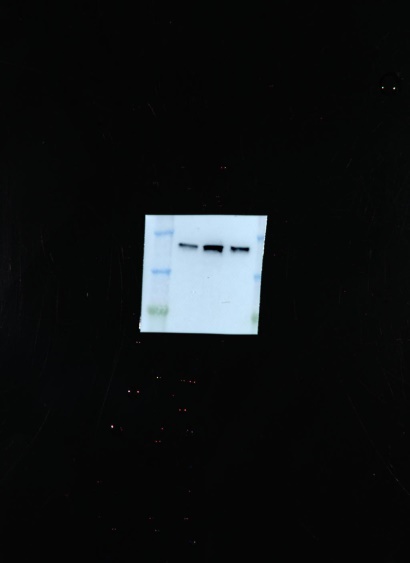

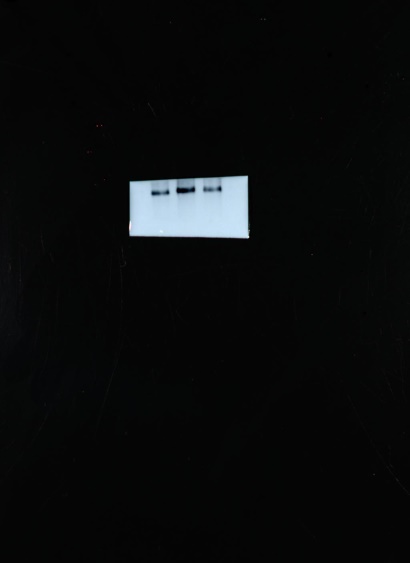


**MMP-9**

**Cyclin D1**

**β-catenin**


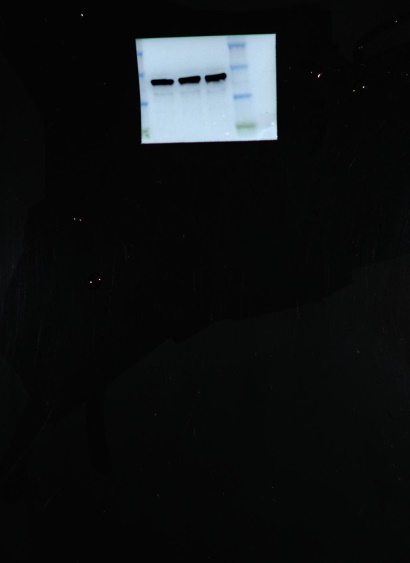


**GAPDH**


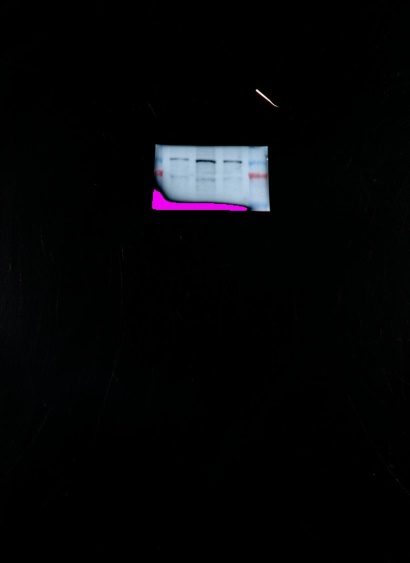

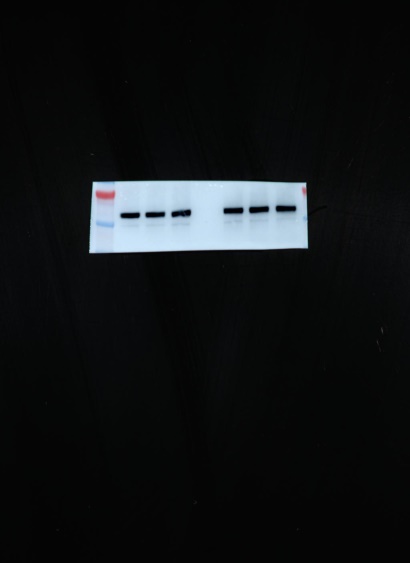


**Fig. 5A：**

**Fig. 4E：**

**GAPDH**

**β-catenin**

| Control | E2 | E2+calpeptin | E2+Super-TDU | E2+Super-TDU |
| --- | --- | --- | --- | --- |
| 100 | 124.3 | 102.1 | 105.2 | 98.2 |
| 100 | 119.5 | 96.7 | 100.1 | 99.6 |
| 100 | 129.9 | 104.5 | 103.3 | 99.3 |

**Fig. 5B：**


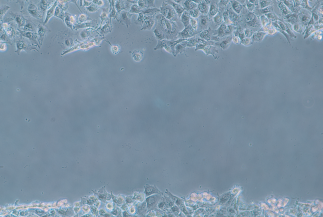

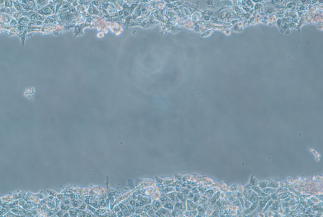

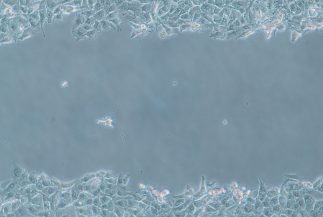

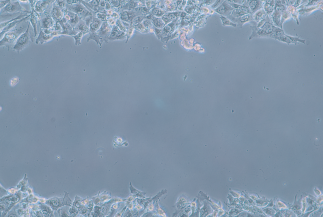

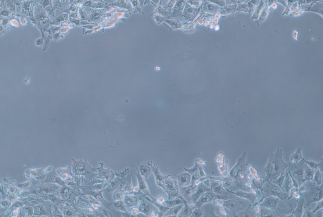

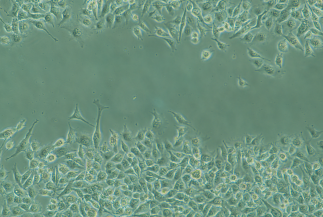

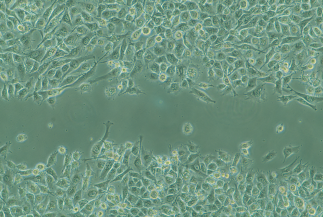

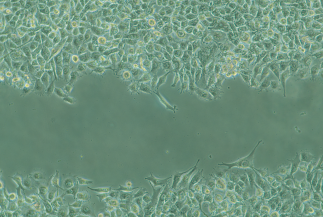

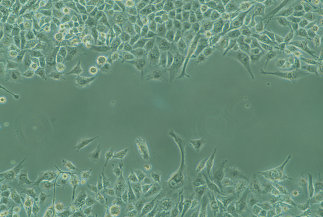

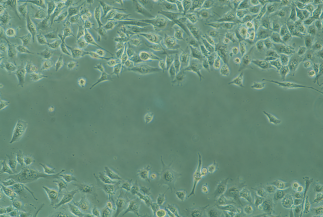


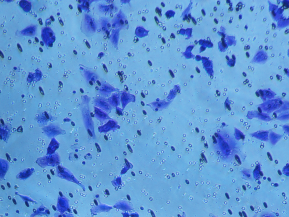

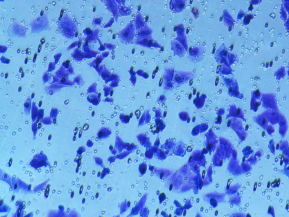

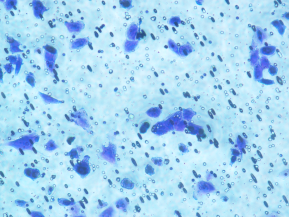

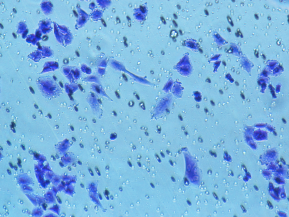

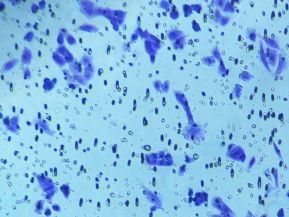


**Fig. 5C**

**Fig. 6A**


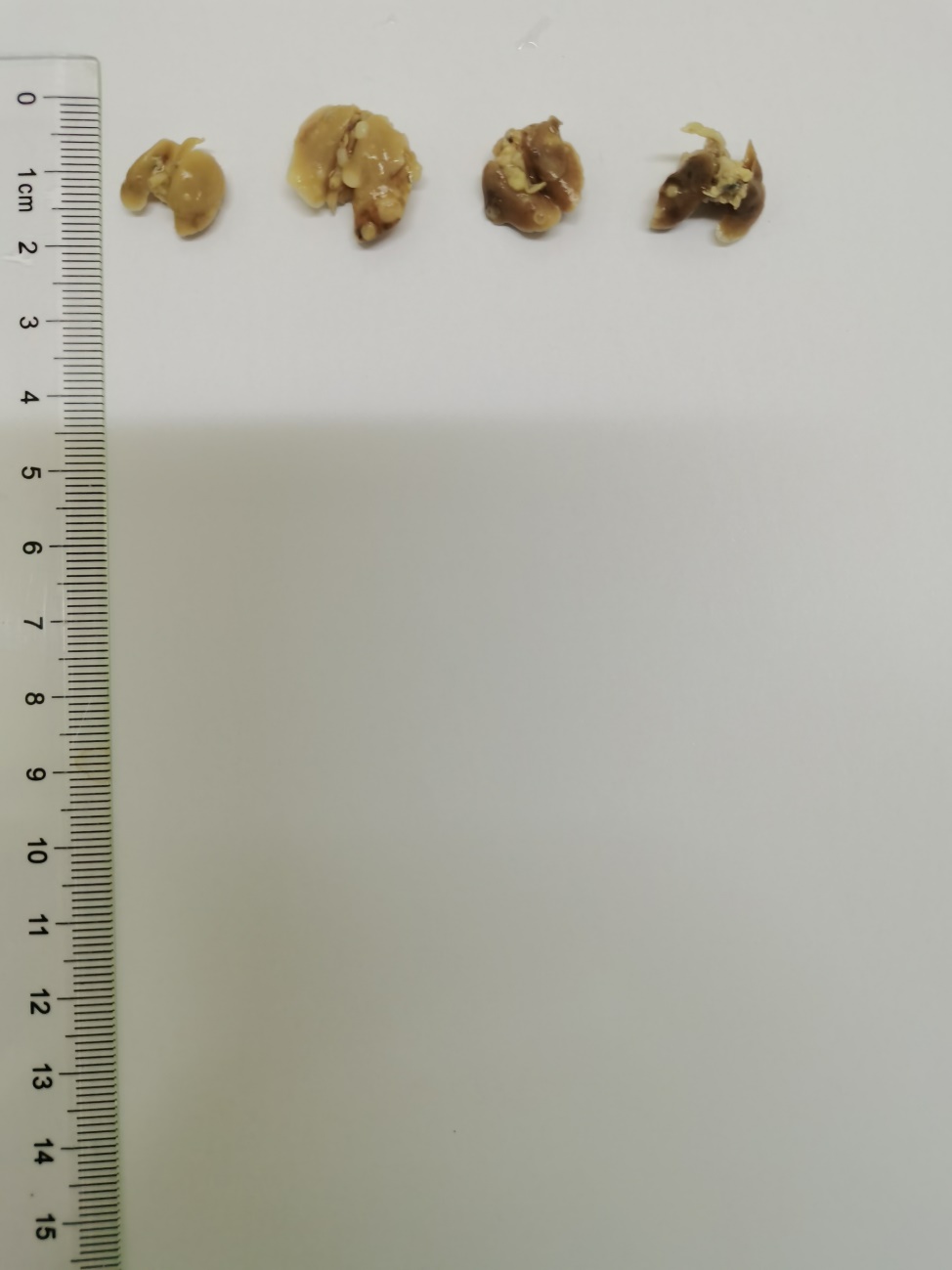


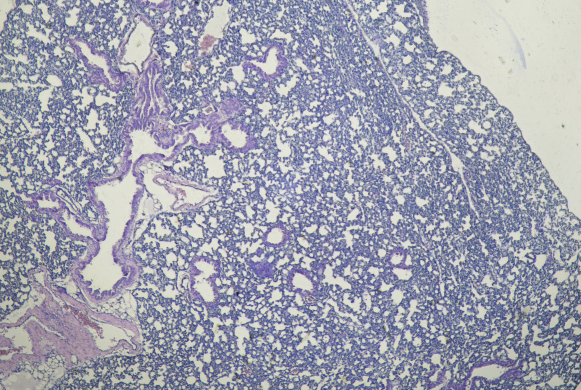

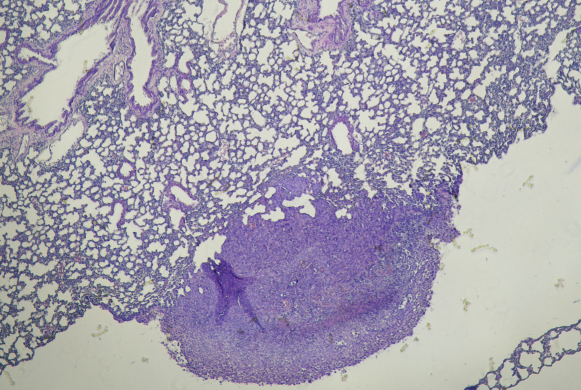

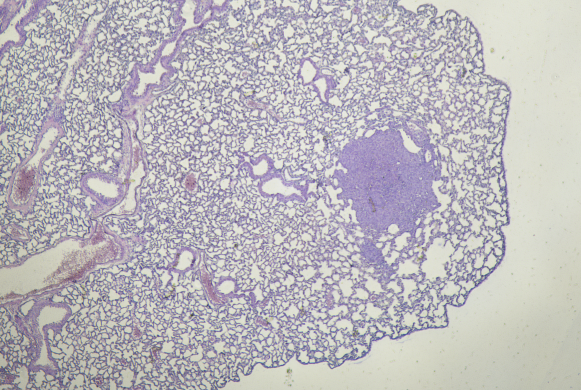

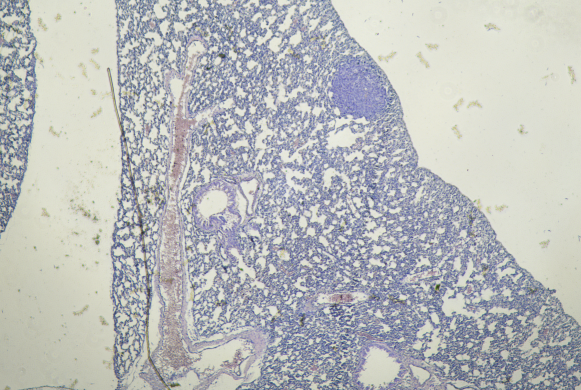


**Fig. 6B**
